# A mild and highly effective method of leaching metal from chalcopyrite using household chemicals

**DOI:** 10.1039/d6ra00565a

**Published:** 2026-03-25

**Authors:** Zhengkun Fang, Paul N. Duchesne

**Affiliations:** a Department of Chemistry, Queen's University Kingston Ontario Canada paul.duchesne@queensu.ca

## Abstract

While chalcopyrite is the most abundant natural source of Cu on Earth, its chemical inertness hinders the practical accessibility of this copper. Many current novel leaching methods rely on using relatively hard (and toxic) compounds (*e.g.*, H_2_SO_4_ and ethylene glycol) and/or reaction conditions to overcome this inertness; however, this results in significant operational hazards and environmental pollution. To address these concerns and in accordance with Green Chemistry principles, we report an environmentally benign leaching system using acetic acid and hydrogen peroxide that enables complete copper recovery at room temperature. Moreover, an environmentally benign planetary ball milling pretreatment was used to degrade the chemical inertness of chalcopyrite in a sustainable way without the need for additional chemical reagents. Ball-milling speed, acetic acid volume, reaction temperature, and reaction duration were varied to achieve optimal leaching conditions for both environmental friendliness and energy efficiency. Consequently, a 100% copper recovery rate was achieved under optimal conditions. Notably, neither inhibition of the leaching nor passivation of the chalcopyrite was observed when employing this leaching system. Thus, this leaching system offers significant advantages in both safety and efficacy for the leaching of copper from chalcopyrite.

## Abstract 2

Through Taguchi experimental design, 100% copper metal recovery from recalcitrant ores is achieved using greener reagents and mild operating conditions, highlighting the role of radical species in hydrometallurgy.

## Graphical abstract

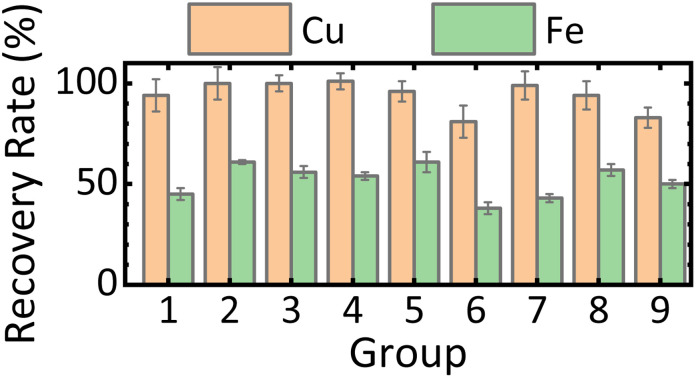

## 1. Introduction

Chalcopyrite (CuFeS_2_), the dominant natural form of copper (Cu), constitutes 70 wt% of the global Cu supply,^1–8^ with most such ores having low Cu concentrations (<0.5 wt%).^4^ In nature, CuFeS_2_ tends to form alongside other sulfide minerals, such as pyrite (FeS_2_), sphalerite (ZnS), and galena (PbS).^9,10^ Both the Cu and iron (Fe) contained in CuFeS_2_ are indispensable to daily life and research. In particular, the superior conductivity and corrosion resistance of Cu are vital in electronic equipment manufacturing and building construction.^11^ Likewise, Fe is an indispensable precursor for all steels and ferritic magnetic materials. From a research perspective, both Cu and Fe also express excellent catalytic activity in specific applications, including wastewater treatment and CO_2_ conversion.^12,13^ However, due to the high heat resistance and chemical inertness of CuFeS_2_, both traditional pyrometallurgical and hydrometallurgical processes tend to work inefficiently.^1,4–7,14,15^

Pyrometallurgy, the oldest metallurgical technique, has been utilized and developed for over 6000 years, with its earliest recorded implementations dating to the Bronze Age.^3,7,16^ Pyrometallurgical processes involve the thermal treatment of minerals with reactive elements, with common examples including calcination using oxygen (O_2_) and smelting using carbon (C) or hydrogen (H_2_).^3,7^ Despite its widespread industrial use, such methods demand a minimum concentration of *ca.* 20 wt% of the desired metal in the mineral, limiting their usefulness for the great majority of CuFeS_2_ ores.^7,17^ This drawback arises mainly from the inherently high refractoriness of these low-grade ores, which requires great energy input to yield the same yields as high-grade ores.^16^ Furthermore, pyrometallurgy operates at elevated temperatures (typically 500 to 2000 °C), resulting in intense energy consumption and significant working hazards.^11^ Finally, pyrometallurgy generates harmful gases (*e.g.*, H_2_S, SO_*x*_, and CO) that contribute to environmental pollution, especially when sulfur-containing minerals are utilized.^7^ Given these numerous shortcomings and challenges, further optimizing the pyrometallurgy method is far from trivial.

In comparison to pyrometallurgy, hydrometallurgy has recently drawn greater research attention by virtue of its relatively low environmental impact and its ability to effectively recover metals from low-grade ores.^7^ Unlike pyrometallurgical processes, hydrometallurgical processes run under much milder temperature conditions (*e.g.*, 25 to 250 °C).^4–7^ Hydrometallurgical processes extract metals from minerals *via* chemical leaching, dissolving minerals directly into solution. Given that the solubility of metal varies at different pH levels, the vast majority of hydrometallurgical methods employ pH-modifying agents. Because the vast majority of metals dissolve readily under acidic conditions, the acidification reagents are implemented to achieve non-selective metal leaching.^4,8,18^ Conversely, alkaline compounds are used to achieve the selective extraction of specific metals.^4,19^ However, the pH-modifying reagent along typically results in very limited metal leaching efficiency from CuFeS_2_.

For example, Medina Ferrer *et al.* previously compared the dissolution of CuFeS_2_ with that of chalcocite (Cu_2_S) in 3.0 M ammonia at 25 °C for 40 min^15^ Under these conditions, roughly 15% of the Cu present was leached from Cu_2_S; however, only minimal Cu was released from CuFeS_2_. Perhaps for this reason, many publications also emphasize the importance of oxidative potential within the leaching solution in the extraction of Cu from this mineral.^1,4–7,14,20^ By deliberately introducing oxidizing agents and tuning oxidative potential, the leaching solution can more effectively break the metal–sulfur bond. Additionally, the oxidation of S^2−^ ions to elemental sulfur (S) or anionic sulfur compounds (*e.g.*, SO_4_^2−^) ensures a high metal recovery rate by preventing the reprecipitation of Fe or Cu ions by S^2−^. In addition to initiating the leaching reaction, oxidizing agents are beneficial in avoiding the generation of air pollutants (*e.g.*, *via* the complete oxidation of H_2_S and SO_*x*_ gases to SO_4_^2−^ ions). While both Fe^3+^/Fe^2+^ couples and H_2_O_2_ are commonly used oxidizing agents in CuFeS_2_ leaching, this research focuses on H_2_O_2_ due to its strong oxidative potential and non-polluting properties.^3,4,6,8^

On the other hand, a diverse range of assistive compounds is often introduced to enhance the leaching performance of CuFeS_2_. Reducing agents (*e.g.*, metallic Fe) can be introduced to convert CuFeS_2_ to less recalcitrant species, such as Cu_2_S, that are more reactive to subsequent leaching.^8,18,20,21^ More unorthodox additives, such as fungi, are detailed by Barton & Hiskey^4^ and Li *et al.*^8^ Additionally, ethylene glycol (EG) or similar compounds may be added to chelate Cu and Fe ions, thereby weakening their catalytic decomposition of H_2_O_2_ in solution and increasing the effectiveness of overall metal leaching.^22–24^

In addition to efficiently leaching metals from CuFeS_2_, it is also necessary to address the high toxicity and environmental impact of these leaching reagents. Although diluting the aforementioned reagents can help mitigate operational safety hazards, diluted compounds are less readily available, more expensive to purchase, and generating more waste, thus undermining efforts to build an economically and environmentally friendly leaching environment. Therefore, it is best to focus first on using reagents that are safe and sustainable. In pursuit of this objective, two key concepts have been integrated into this study.

Firstly, mechanical ball milling is employed to overcome the inherent chemical inertness of CuFeS_2_ ore, instead of chemically converting it to more reactive compounds. The increased reactivity can be attributed to altering particle size and/or introducing defects to the original crystal structure.^1,7,25–27^ Overall, this solid-state mechanical treatment eliminates the use of any other chemicals and solvents, fulfilling Green Chemistry principles 1 (waste prevention), 3 (less hazardous chemical synthesis), and 5 (safer solvents & auxiliaries).

Second, concentrated acetic acid (CH_3_COOH, the principal component of vinegar) is used instead of harsh acids (*e.g.*, H_2_SO_4_) to reduce the working hazard and toxicity. This is possible because CH_3_COOH (p*K*_a_ = 4.7) is more acidic than hydrogen sulfide (H_2_S, p*K*_a_ = 7.0), allowing it to break metal–sulfur bonds in CuFeS_2_ by protonating S^2−^. In addition, the acidic environment created by CH_3_COOH increases both the oxidation ability and stability of oxidizing agents, which is especially important for H_2_O_2_. Moreover, the use of concentrated CH_3_COOH satisfies Green Chemistry principles 1 (waste prevention), 3 (less hazardous chemical synthesis), and 12 (inherently safer chemistry for accident prevention).

The utilization of CH_3_COOH in hydrometallurgy, such as galena leaching, has been discussed in multiple reports;^28^ however, there are few reports of CH_3_COOH use in leaching CuFeS_2_. Solís-Marcial and Lapidus first reported in 2014 that adding CH_3_COOH into the H_2_SO_4_/O_3_/CuSO_4_ system could significantly increase the CuFeS_2_ dissolution rate and efficiency.^29^ Later, Chen *et al.* were the first to use CH_3_COOH–H_2_O_2_ with the assistance of NaCl to achieve an almost 100% Cu recovery rate from CuFeS_2_.^30^ Despite the priority on using CH_3_COOH to achieve high-efficiency CuFeS_2_ leaching, they did not provide clear and convincing explanations of the accessibility of this combination and the effect of leaching conditions on the leaching performance. Therefore, it is essential to perform thorough research to verify the validity of this combination of reagents.

In this study, the feasibility of using CH_3_COOH/H_2_O_2_ to leach metal from CuFeS_2_ is first assessed. Next, the effects of ball milling speed, CH_3_COOH volume, leaching temperature, and leaching time on metal recovery from CuFeS_2_ were analysed based on the inductively coupled plasma-optical emission spectroscopy (ICP-OES), powder X-ray diffraction (PXRD), X-ray photoelectron spectroscopy (XPS), and Taguchi analyses. Eventually, based on these results, optimal set of reaction conditions is proposed for CuFeS_2_ leaching, and insights are offered into the mechanisms underlying this reaction.

## 2. Experimental

### 2.1 Materials and methods

This study employed reagent-grade chemicals, including acetic acid (CH_3_COOH, ≥99.7 wt%), hydrogen peroxide (H_2_O_2_, 30 wt%), and sodium hydroxide solid (NaOH, 97 wt%), purchased from Millipore-Sigma. Reagent-grade hydrochloric acid (HCl, 36.5–38.0 wt%) was purchased from Fisher Scientific. ACS-grade nitric acid (HNO_3_, 68.0–70.0 wt%) was purchased from Anachemia. Distilled water was obtained from laboratory supply.

The as-obtained CuFeS_2_ ore particle size ranged from 0.125 mm to 4 mm, confirmed by using laboratory sieves. The chemical composition of the Ore, obtained using aqua regia digestion followed by ICP-OES analysis, was 1.24 ± 0.12 wt% Cu, 18.6 ± 1.4 wt% Fe, and 4.58 ± 0.29 wt% Zn. Reported concentrations are the mean of three independent ICP-OES replicates, with the error indicating the standard deviation (1*σ*). According to PXRD analysis, as shown in Fig. S1, Cu is present predominantly in chalcopyrite; Fe exists in chalcopyrite and pyrite; and Zn is primarily found in a sphalerite phase. Other major components included quartz, clinochlore, and muscovite.

### 2.2 Experimental

The ore was dry milled in a Retsch planetary ball mill (PM100) with 10 mm diameter zirconium dioxide balls (*m*_ore_ : *m*_ball_ = 1 : 5). The ball mill was operated at a fixed milling speed (300 rpm, 400 rpm, or 500 rpm) for 1 h. For brevity, “BM-300”, “BM-400”, and “BM-500” are used to signify ores milled at 300 rpm, 400 rpm, and 500 rpm, respectively. Next, roughly 1.5 g of milled ore was transferred to the leaching reactor, as shown in Fig. S2. A NaOH safety bottle (Fig. S2a) was used to prevent the potential emissions of SO_*x*_ or H_2_S gases from the reactor. Experimental design and controlled parameters, including leaching temperature, leaching time, ball milling speed, and the volume of CH_3_COOH, are shown in Table 1. Distilled water was added to ensure a consistent mass concentration of 1 g ore per 20 mL solution for all groups. A 600-rpm stirring speed and 15 mL of 30 wt% H_2_O_2_ were used for all groups.

**Table 1:** Design of experiment (L9 orthogonal array)

Taguchi design – L9 orthogonal array				
Experiment	Milling speed (rpm)	Leaching temperature (°C)	Volume of 17 M CH_3_COOH (mL)	Leaching time (h)
1	300	RT (∼20)	5	3
2	300	40	10	4
3	300	60	15	5
4	400	RT (∼20)	10	5
5	400	40	15	3
6	400	60	5	4
7	500	RT (∼20)	15	4
8	500	40	5	5
9	500	60	10	3
8	500	40	5	5
9	500	60	10	3

The minimum addition amounts of CH_3_COOH and H_2_O_2_ were determined using the ore itself as a limiting reagent. By assuming that the ore was a pure substance of each of its three main components (CuFeS_2_, FeS_2_, and ZnS), the maximum consumption of CH_3_COOH and H_2_O_2_ was calculated, providing the basis for the minimum addition amount. To ensure sufficient reagent availability despite undesired side reactions (*e.g.*, H_2_O_2_ decomposition), the amount of CH_3_COOH and H_2_O_2_ used in this research was two to five times greater than this minimum value.

Following the leaching, vacuum filtration was employed to separate the ore residue (*i.e.*, the solid material remaining in the reactor post-leaching) from the leachate. The leachate was then dried under air at 200 °C overnight, while the ore residue was air dried at room temperature (*ca.* 20 °C) overnight.

After quantifying the metal concentration in the leachate, the metal recovery rate was calculated using eqn (1).1
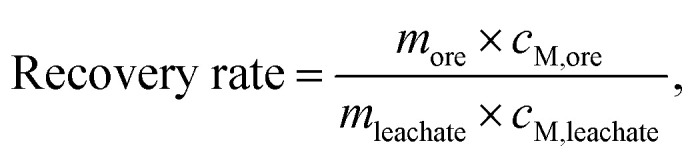
wherein *m*_ore_ is the initial mass of ore added to the reactor, *m*_leachate_ is the mass of leachate, *c*_M,ore_ is the concentration of metal (Cu or Fe) in the ore, and *c*_M,leachate_ is the concentration of metal (Cu or Fe) in the leachate.

Subsequently, Taguchi data processing is built upon each group's signal-to-noise (S/N) ratio.^31–33^ Since the highest recovery rate is desired in this study, the “larger value is better” equation is used. The expression of the equation could be formulated as follows in eqn (2):2
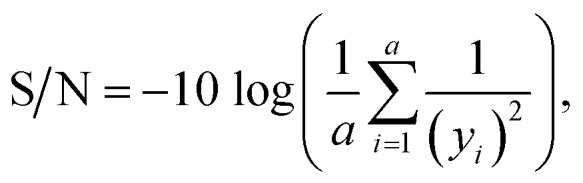
where *a* is the number of runs per group and *y*_*i*_ is data value (in this case, the metal recovery rate) for the *i*-th run.^31^ The S/N ratio of each level is determined by averaging the S/N ratio of the groups associated with that level. Finally, range (*R*, calculated by subtracting the factor's smallest S/N ratio from its largest S/N ratio) could be used to determine the relative impact of each factor on the leaching reaction.

### 2.3 Characterization

PXRD measurements were performed at the Queen's Facility for Isotope Research (QFIR) using a Malvern Panalytical Empyrean diffractometer. Ore samples were ground to a fine, uniform powder in a quartz mortar and packed into sample holders prior to analysis. Samples were analysed at 2*θ* angles ranging from 5 to 100° with a dwell time of 180 s using Cu Kα radiation (*λ* = 1.54 Å). The X-ray tube was operated at a voltage of 45 kV and a current of 40 mA. Mineral identification was performed using Highscore Pro 4.9 software and the International Center for Diffraction Data PDF-4 + 2024 database.^34,35^

Particle sizes of milled ores were measured using a Mastersizer 3000 from Malvern Panalytical and a Hydro LV dispersion unit. The milled samples were first dispersed in 100 mL of 0.5% sodium hexametaphosphate solution and left for 24 hours. Before measurement, solutions were sonicated for 10 min to deflocculate the suspended material. Each sample was measured in triplicate. Samples were analysed as non-spherical particles with a presumed refractive index of 1.6 and a density of 4.2 g mL^−1^.

ICP-OES was performed at QFIR using a Thermo Scientific iCAP PRO Series ICP-OES coupled with a 4DX Elemental Scientific prepFAST M5 autosampler for sample introduction and dilution. Ore samples were digested in aqua regia solution (VHNO_3_ to VHCl ratio = 1 : 3), whereas leaching products were digested in concentrated HNO_3_. The entire digestion was performed at 110 °C until near dryness, following which digested samples were diluted to 100 ppm with 2 vol% HNO_3_.

ICP-OES operating conditions and sample introduction information are shown in Table 2. Calibration curves were calculated using single-element Spex CertiPrep Claritas PPT standards diluted to a 2 to 100 µg L^−1^ range. Reference material CCu-1c, certified by the Government of Canada, and blanks were measured to determine background concentrations, instrument drift, and verify results.

**Table 2:** Operating conditions for ICP-OES

Parameter	Operating conditions
Spray chamber	Cyclonic (Thermo Scientific)
Nebulizer flow	0.5 L min^−1^
Plasma view	Axial
Measurement mode	iFR (167.021 to 852.145 nm)
Exposure time	2 s
Sample repeats	15

XPS spectra were measured on a Kratos Axis Nova spectrometer equipped with an Al Kα X-ray source (1486.69 eV, 150 W, 10 mA), a charge neutralizer, and a delay-line detector (DLD) consisting of three multi-channel plates. Tests were performed in an ultrahigh vacuum analysis chamber (10^−9^ Torr), and spectra were measured in the range of −5 to 1200 eV with a pass energy of 160 eV (scans: 1, dwell time: 100 ms) and an energy step size of 1 eV. The incident angle (X-ray source to sample) was the magic angle (54.75°), and the take-off angle (sample to detector) was 90 °C.

In preparation for XPS testing, samples were first ground to a fine powder in a quartz mortar and then affixed to an SEM mount (with pins cut off) using double-sided adhesive Cu tape. Next, loose powder was removed using compressed air, followed by application of a second double-sided adhesive Cu tape to affix the SEM mount to a coated aluminum plate. Samples were left in the preparation chamber under high vacuum (10^−8^ Torr) overnight prior to measurement.

XPS peak fitting was performed in CasaXPS 2.3.26,^36^ with the photoelectron binding energy was calibrated by assigning the C 1s C–C/C–H peak an energy of 284.4 eV. A Shirley background and blended Gaussian–Lorentzian peak shapes were used to fit all spectra.

Ultraviolet-visible (UV-vis) absorption spectra were recorded at room temperature using a PerkinElmer Lambda XLS UV/VIS absorbance spectrometer. All sample solutions were placed in standard quartz cuvettes with an optical path length of 1 cm. Prior to sample analysis, a baseline correction was performed using MilliQ water as the blank reference. Absorption spectra were collected across a wavelength range of 200 to 900 nm.

## 3. Results

### 3.1 Feasibility of the CH_3_COOH/H_2_O_2_ system

Control experiments were first conducted to test the feasibility of the combined CH_3_COOH/H_2_O_2_ leaching system for CuFeS_2_ and ascertain the role of each component. Yields in this section are reported based on the mass of precipitated leached recovered from 1 g of ore. The presence of Cu and Fe in the product is supported by qualitative observations presented in Fig. S3. Notably, a red-brown precipitate was acquired after adding NaOH to the leachate, indicating the presence of Fe^3+^ ions. A blue colour was also observed upon increasing the pH of the leachate solution to 12, highlighting the presence of Cu^2+^ ions.

**Fig. 1:**
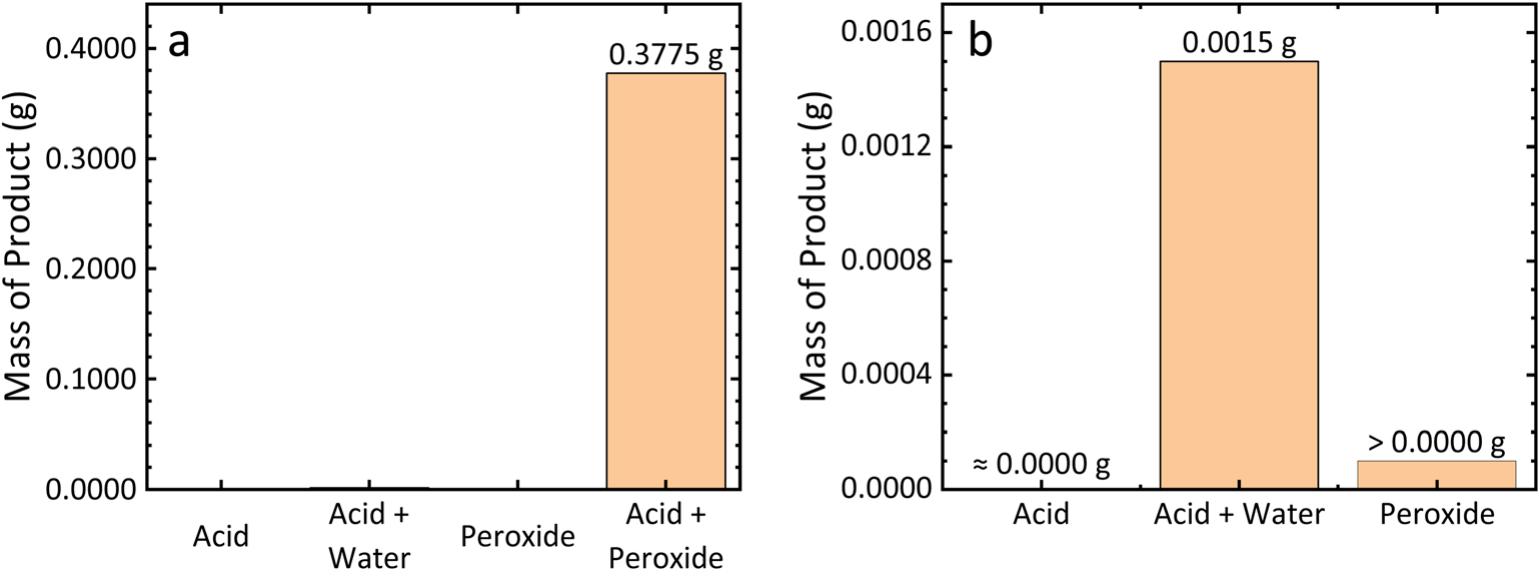
(a) Demonstration of the requirement for both CH_3_COOH and H_2_O_2_ used in combination, and (b) magnification of the three ineffective treatment solutions (acid, acid + water, and peroxide) groups.

Fig. 1a and b show that no product was obtained using CH_3_COOH alone (*i.e.*, “acid”). Notably, the high concentration of 17 M CH_3_COOH results in a minimal concentration of H^+^ ions, due to its limited ability to auto-dissociate, explaining the low yield of this test. To investigate whether this was a limiting factor, CH_3_COOH was also diluted with distilled water (“acid + water”) to encourage its ionization, resulting in a very small quantity (0.0015 g) of obtained product. In both of these cases, re-precipitation of metal sulfides and the minimal oxidizing ability of CH_3_COOH limit its reactivity.

Next, testing H_2_O_2_ alone (“peroxide”) yielded a trace amount of products (visible in the reaction vessel); however, the quantity so obtained was too small to register on a standard laboratory balance. Notably, a vigorous H_2_O_2_ decomposition occurred within 1 h of beginning the leaching reaction, indicated by the rapid generation of abundant bubbles. Finally, when combining both CH_3_COOH and H_2_O_2_ (“acid + peroxide”), 0.3775 g of product was obtained, with the H_2_O_2_ visibly reacting over the course of at least 4 h.

It can thus be concluded that the CH_3_COOH/H_2_O_2_ combination is capable of leaching metal from CuFeS_2_ ore with H^+^ ions and H_2_O_2_ being the key reagents. Here, water present in the added H_2_O_2_ solution facilitates the generation of H^+^ by promoting CH_3_COOH dissociation. Additionally, compared with the peroxide group, the acid + peroxide group revealed markedly lower H_2_O_2_ decomposition, allowing for greater H_2_O_2_ participation in CuFeS_2_ leaching. Thus, suggests that, in addition to directly protonating sulfide to H_2_S, H^+^ ions could significantly inhibit H_2_O_2_ decomposition. These highlight the synergistic effect between H^+^ ions and H_2_O_2_ in enhancing CuFeS_2_ leaching efficiency.

### 3.2 Taguchi analysis

**Fig. 2:**
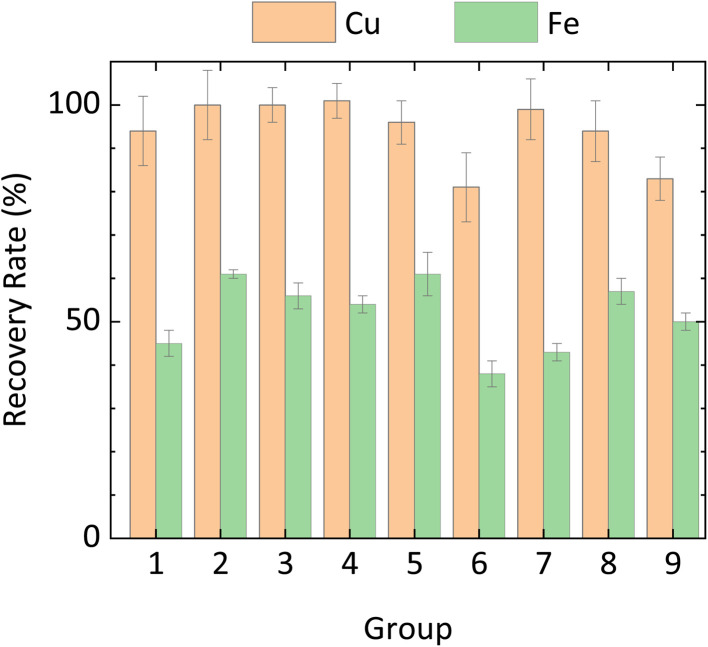
Averaged Cu and Fe recovery rates for each group used in the Taguchi analysis.

Having confirmed the feasibility of the CH_3_COOH/H_2_O_2_ system, the optimal leaching conditions were explored in detail. Four key factors (ball milling speed, CH_3_COOH volume, leaching time, and leaching temperature) were selected based on the results of preliminary experiments. Cu and Fe recovery rates, obtained for each parameter combination in the Taguchi analysis and averaged across three sets of experiments, are shown in Fig. 2. Recovery rates exceeding 100% reflect an inherent inhomogeneity in the mineral material that was not resolved by the processing methods employed in this study. Error bars indicated the standard deviation (1*σ*) of metal recovery under each set of reaction conditions. The relative standard deviation across all ICP-OES measurements was consistently lower than 3.6%. Note that the Fe recovery rate includes leaching from both FeS_2_ and CuFeS_2_.

Table 3 shows the depth analysis results of orthogonal Taguchi experiments and the predicted optimal leaching conditions for Cu and Fe based on their S/N ratio. Among all experimental parameters, leaching temperature had the greatest influence on both Cu and Fe recovery, followed by CH_3_COOH volume, leaching time, and, finally, milling speed. The optimal combination of these four factors for Cu recovery was found to be: BM-300 particles, 15 mL of 17 M CH_3_COOH, and leaching at room temperature for 5 h. Similarly, those for optimal Fe recovery were BM-300 particles, 10 mL of 17 M CH_3_COOH, and leaching at 40 °C for 5 h. To validate these results, CuFeS_2_ leaching was performed three times under each of these two optimal conditions. ICP-OES was used to analyse Cu and Fe concentrations in the resulting leachates, and PXRD was used to characterize the ore residue collected after the leaching reaction.

**Table 3:** Deep analysis of orthogonal experiments for Cu and Fe

Level	Milling speed (rpm)	Leaching temperature (°C)	Volume of acetic acid (mL)	Leaching time (h)
**Cu**				
1	39.74	39.80	38.98	39.15
2	39.29	39.60	39.41	39.27
3	39.21	38.84	39.86	39.82
Maximum value	39.74	39.80	39.86	39.82
Minimum value	39.21	38.84	38.98	39.15
Range	0.52	0.96	0.88	0.67
Rank	4	1	2	3
Optimal	BM-300	RT	15	5

**Fe**				
1	34.56	33.44	33.16	34.22
2	33.91	35.46	34.80	33.27
3	33.91	33.48	34.43	34.90
Maximum value	34.56	35.46	34.80	34.90
Minimum value	33.91	33.44	33.16	33.27
Range	0.65	2.02	1.65	1.63
Rank	4	1	2	3
Optimal	BM-300	40	10	5

**Fig. 3:**
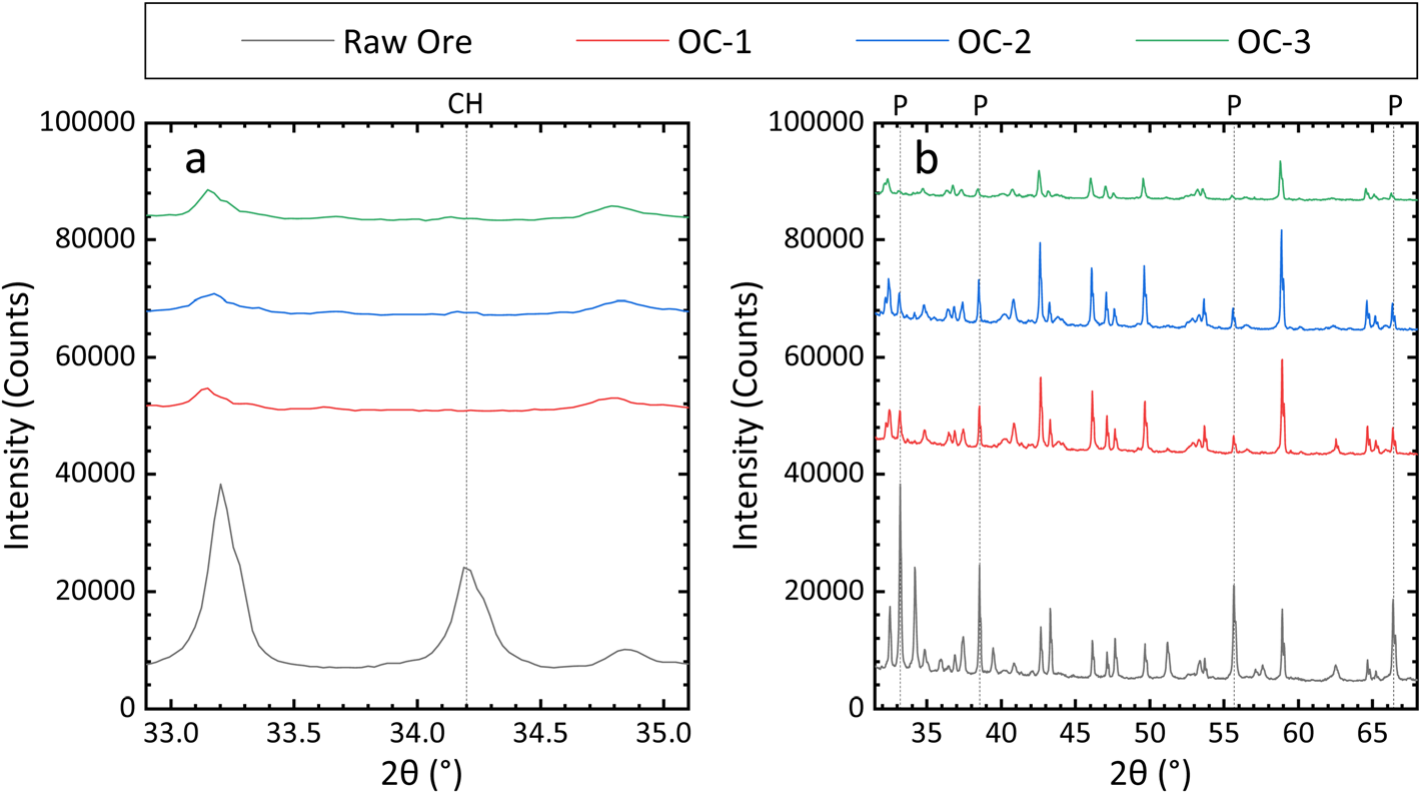
The PXRD pattern of the ore residue under optimal leaching conditions for (a) Cu and (b) Fe, each performed in triplicate. (OC: optimal condition; CH: CuFeS_2_; P: FeS_2_.)

Impressively, a 100 ± 8% Cu recovery rate was achieved under Cu recovery optimal conditions, and no CuFeS_2_ peak was visible in the PXRD pattern of the ore residue (Fig. 3a and S4a).

**Fig. 4:**
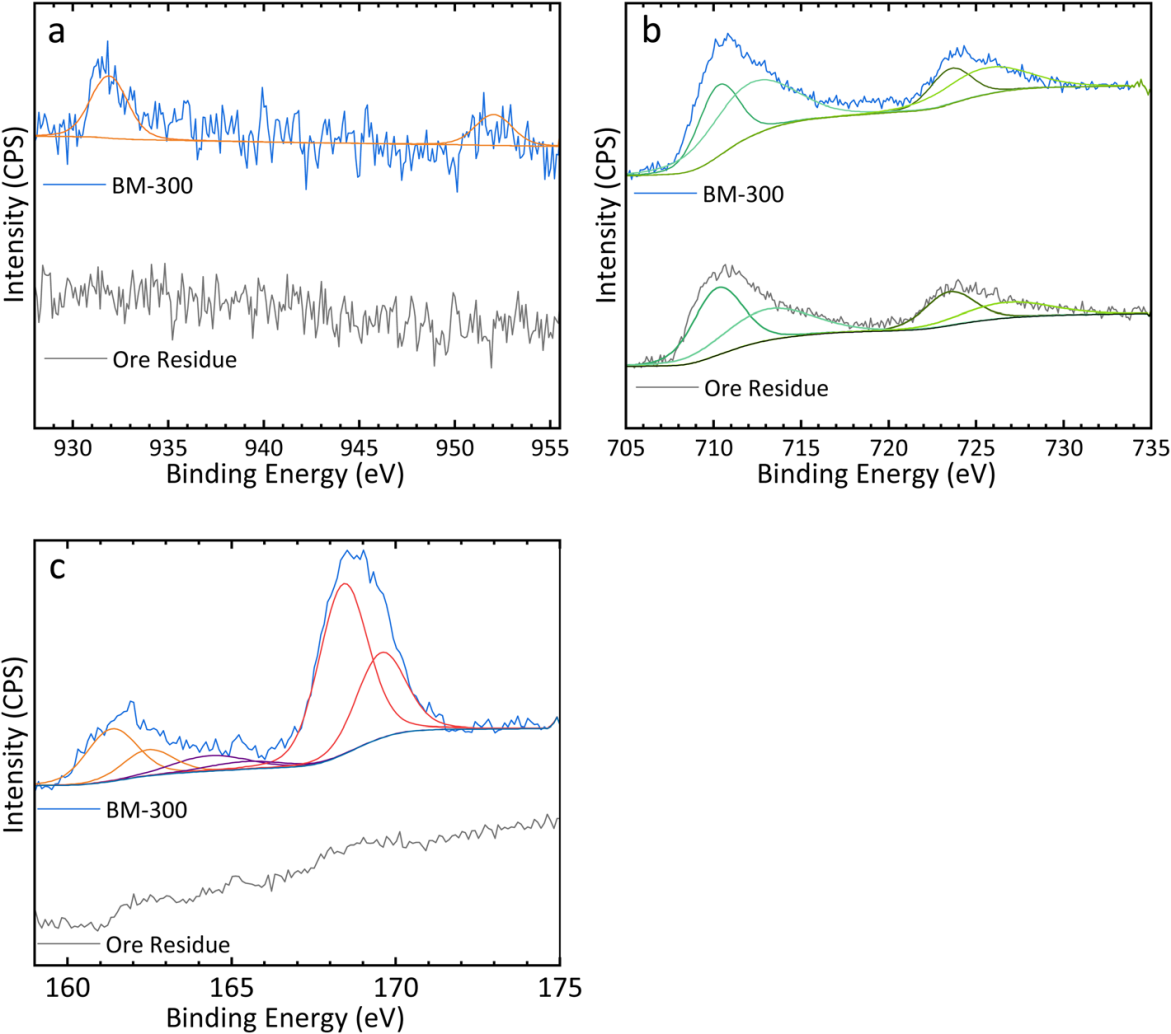
(a) Cu 2p, (b) Fe 2p, and (c) S 2p XPS spectra obtained from both BM-300 particles and ore residue leached under Cu recovery optimal leaching conditions.

The Fe recovery rate, obtained under Fe recovery optimal conditions, was 55 ± 2%. Again, the PXRD results further corroborated those from ICP-OES. Most Fe in the ore exists in the form of FeS_2_; therefore, regarding Fe recovery, the main focus should be placed upon the intensities of characteristic FeS_2_ peaks. As shown in Fig. 4b and S4b, relative to that of the raw ore, the characteristic FeS_2_ peak intensity in the ore residue significantly decreased following leaching.

Unexpectedly, the recovery rate of Fe under optimal conditions is smaller than the highest observed recovery rate (Experiment 4) in the orthogonal array (61 ± 1%). However, the heterogeneous distribution of Fe in the ores alone (having a variation of 1.37 wt%) could result in as much as 4% uncertainty in the Fe recovery rate. By considering additional experimental sources of uncertainty (*e.g.*, temperature fluctuations and the inherent uncertainty of the analytical balance), it can be concluded that the difference between the two sets of conditions is within acceptable error.

XPS was performed to analyse both the raw ore and ore residue, in order to ascertain the unique benefits of the CH_3_COOH/H_2_O_2_ leaching system. Fig. 4 shows the Cu 2p, Fe 2p, and S 2p spectra of the ore residue of Cu–OC1 and BM-300 particles, while Table 4 shows the peak-fitting parameters for each element. The Cu 2p spectrum for the BM-300 particles was fitted using a pair of peaks at 931.9 and 952.0 eV with a 2 : 1 areal ratio between Cu 2p_3/2_ and 2p_1/2_ and identical full width at half-maximum (FWHM) values. This paired Cu peak analysis is consistent with the Cu 2p peak in previous XPS studies of CuFeS_2_.^8,37^ In contrast, no Cu peaks were detectable in the ore residues, corroborating the ICP-OES and PXRD results. A similar phenomenon was observed in the S 2p spectrum; the S 2p peak was fitted with the spin-orbital splitting with a 2 : 1 area ratio and equal FWHM values.^38^ The S 2p spectrum for the BM-300 particles was fitted using three pairs of peaks corresponding to monosulfide (161.4 and 162.5 eV), polysulfide (164.4 and 165.4 eV), and sulfate (168.4 and 169.6 eV), respectively.^8,39^ The sulfate in BM-300 particles is likely due to air oxidation, as it was stored for several weeks without specific precautions to prevent oxidation.^7,8^ No S 2p peaks were detected in the spectrum of ore residue. Additionally, the Fe 2p spectra of both BM-300 particles and ore residue were similar and included both Fe^2+^ (710.3/723.5 eV and 710.2/723.5 eV, respectively) and Fe^3+^peaks (712.1/725.5 eV and 713.1/726.7 eV, respectively); however, the intensity of the peaks in the ore residue was lower than that in the BM-300 samples.

**Table 4:** Peak fitting parameter for the S 2p, Fe 2p, and Cu 2p spectra for BM-300 (left) and ore residue (right). “N/A” indicates that no such peak was observed in the XPS spectrum of this sample

Component	BM-300			Ore residue		
	Binding energy (eV)	FWHM (eV)	Peak area (%)	Binding energy (eV)	FWHM (eV)	Peak area (%)
Monosulfide 2p_3/2_	161.4	1.82	14.69	N/A	N/A	N/A
Monosulfide 2p_1/2_	162.5	1.82	7.34	N/A	N/A	N/A
Polysulfide 2p_3/2_	164.4	2.67	6.34	N/A	N/A	N/A
Polysulfide 2p_1/2_	165.4	2.67	3.17	N/A	N/A	N/A
Sulfate 2p_3/2_	168.4	1.75	45.65	N/A	N/A	N/A
Sulfate 2p_1/2_	169.6	1.75	22.81	N/A	N/A	N/A
Fe(ii) 2p_3/2_	710.3	2.78	26.09	710.2	3.35	36.32
Fe(iii) 2p_3/2_	712.1	5.72	40.51	713.1	6.01	30.28
Fe(ii) 2p_1/2_	723.5	2.78	13.08	723.5	3.35	18.21
Fe(iii) 2p_3/2_	725.5	5.72	20.32	726.7	6.01	15.19
Cu 2p_3/2_	931.9	2.29	75.04	N/A	N/A	N/A
Cu 2p_1/2_	952.0	2.29	24.96	N/A	N/A	N/A

Although the chemical inertness of CuFeS_2_ has been discussed intensively in recent literature, there is no clear consensus as to its origin. In recent discussions, a sulfur-rich passivation layer, formed during leaching, and hindered electron transfer by the semiconducting nature of CuFeS_2_ are considered among the most likely explanations.^1,2,5,7,14,37,40^ However, the ICP-OES and XPS results of the present study show neither a slowing of leaching nor any sign of passivation layer formation in the CH_3_COOH/H_2_O_2_ leaching system. All sulfur content was apparently removed from the ore surface during the leaching, indicating that no sulfur-rich passivation layer formed on the surface. Additionally, the average Cu recovery rate for all tested groups was *ca.* 90%, and the leaching reaction was finished within 5 h, proving that CuFeS_2_ is highly reactive under CH_3_COOH/H_2_O_2_ environments. These observations agree with the observation of Solis-Marcial and Lapidus that no passivation properties were found when introducing CH_3_COOH into their H_2_SO_4_/O_3_ system.^29^ This evidence suggests that CH_3_COOH works in conjunction with H_2_O_2_ to overcome the chemical inertness of CuFeS_2_, though it remains to be determined whether this is due to complexation of metal ions by acetate ions or some other chemical mechanism.

### 3.3 The effect of leaching temperature

Before delving into a detailed analysis, it is worth noting that, because of the exothermic nature of this leaching reaction, the temperature of the solution is expected to increase as it proceeds. The exothermic nature was confirmed by an observation that the oil bath and hotplate at *ca.* 35 °C can heat up the leaching solution to *ca.* 40 °C rapidly.

**Fig. 5:**
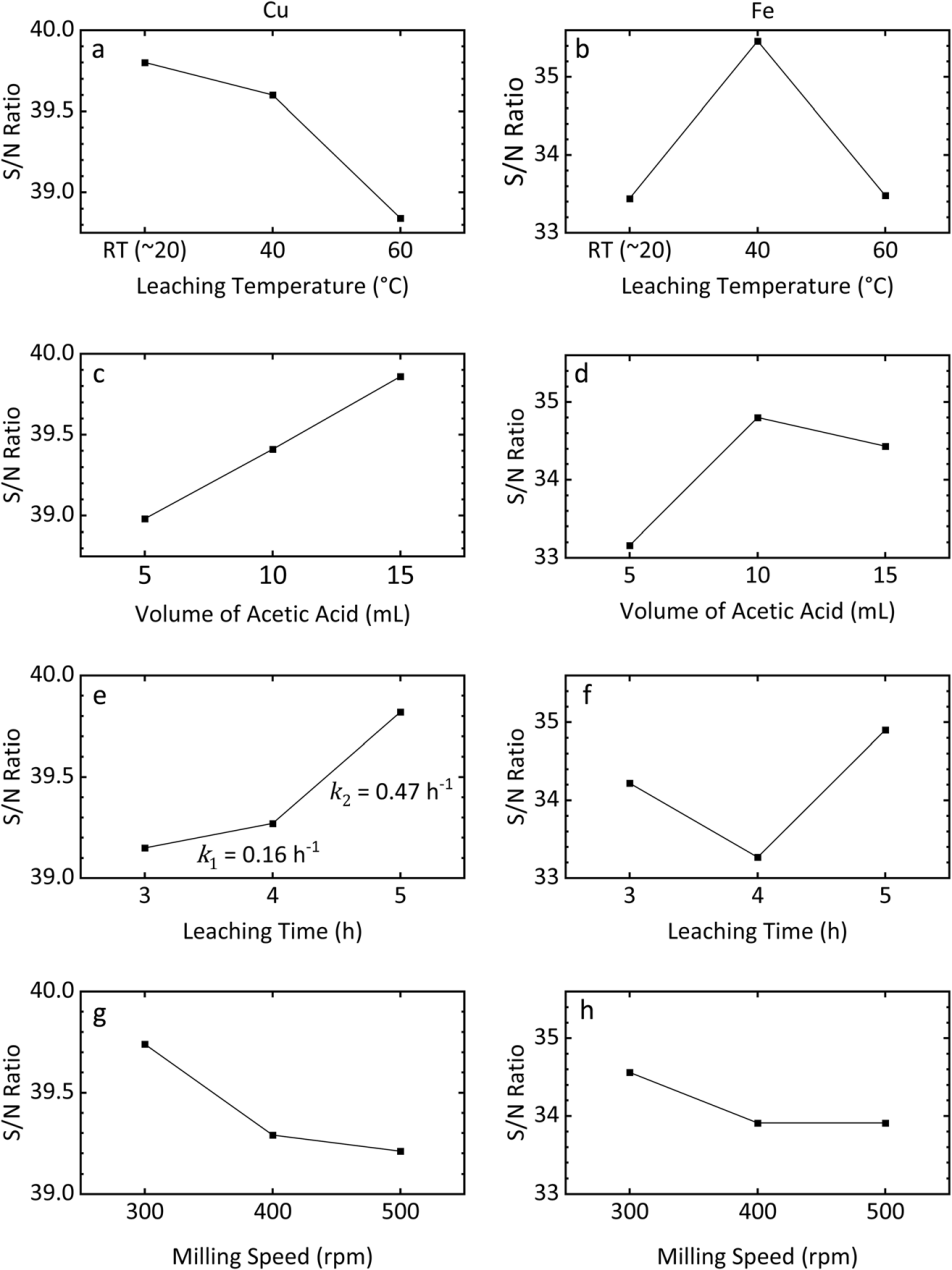
The effect of leaching temperature, CH_3_COOH volume, leaching time, and the milling speed on the recovery rate of (a, c, e, and g) Cu and (b, d, f, and h) Fe recovery.

As illustrated in Fig. 5a and b, the Cu recovery rate was inversely proportional to leaching temperature, whereas the Fe recovery rate increased before decreasing. The changing trend of Fe and Cu recovery rates from RT to 40 °C can be explained by the tendency of FeS_2_ to dissolve at higher temperatures and the catalytic effect of ferric (Fe^3+^) ions on the decomposition of H_2_O_2_, respectively.

**Fig. 6:**
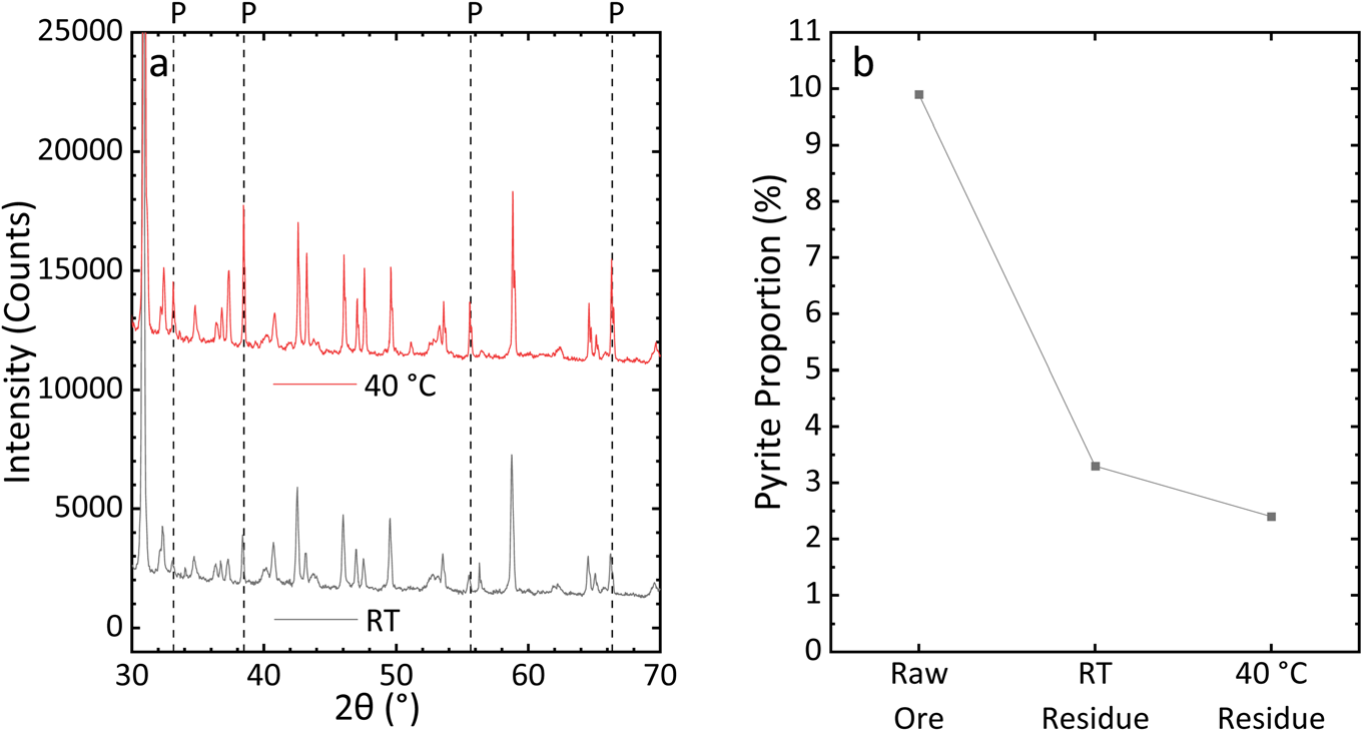
(a) PXRD patterns of ore residue at RT and 40 °C, and (b) the proportion of FeS_2_ in the ore and ore residue after leaching at RT and 40 °C.

FeS_2_ dissolution requires more thermal energy input than CuFeS_2_ dissolution (activation energy of 68 kJ mol^−1^,^41^*versus* 60 kJ mol^−1^ (ref. 42) for CuFeS_2_), explaining an increased Fe recovery rate from RT to 40 °C. To validate this, BM-400 particles were mixed with 15 mL of CH_3_COOH and 15 mL of H_2_O_2_ for 3 h at RT and at 40 °C, and PXRD was performed on the ore residue. As shown in Fig. 6a and S5, the FeS_2_ peak intensity in the ore residue leached at RT is higher than that in the residue leached at 40 °C. The FeS_2_ proportion in un-leached BM-400 particles was measured at 9.9%, but only 3.3% in the ore residue collected at RT, and 2.4% collected at 40 °C.

Additionally, the catalytic effect of Fe^3+^ ions on H_2_O_2_ decomposition can be used to explain the decreasing trend in Cu recovery rate from RT to 40 °C. Previous studies have proven that Fe is dissolved preferentially over Cu and expresses a relatively fast reaction rate.^8,18,43^ This suggests that the leaching system already contains a considerable amount of Fe^3+^ ions when Cu begins dissolving. Increasing temperature further increases the ability of Fe^3+^ ions to catalyse H_2_O_2_ decomposition and causes a substantial amount of H_2_O_2_ to decompose before the dissolution of Cu, explaining a decreased Cu recovery rate from RT to 40 °C.

On the other hand, the observed decrease in recovery rate from 40 to 60 °C for Fe and Cu can be explained by the low thermal stability of H_2_O_2_ in this temperature range. As the temperature approaches 60 °C, H_2_O_2_ decomposes increasingly rapidly, especially under the catalytic action of Fe^3+^ ions. This decomposition results in a H_2_O_2_-deficient environment, thereby impairing the leaching reaction.

### 3.4 The effect of CH_3_COOH volume

Fig. 5c and d show the impact of CH_3_COOH volume on the recovery rate of each element. The Cu recovery rate correlated positively with CH_3_COOH volume, while the Fe recovery performance exhibited a rise followed by a slight fall. As highlighted previously, Fe distribution in the raw ore is heterogeneous. Therefore, it is reasonable to suggest that such a small decrease for Fe could be due, at least in part, to natural variation in Fe abundance within the ore.

Le Chatelier's Principle can be used to explain the effect of CH_3_COOH volume. A simplified reaction equation is used to illustrate this in eqn (3). According to Le Chatelier's Principle, adding more CH_3_COOH drives the reaction toward more complete CuFeS_2_ dissolution. Both sulfite and sulfate ions are included in this equation, as they were detected in sulfur XPS spectra of the leachate in Fig. S6. Pairs of peaks fitted at 168.3 and 169.5 eV, and 168.9 and 170.0 eV correspond to sulfite and sulfate ions, respectively.

3 2CuFeS_2_ + 2CH_3_COOH + 15H_2_O_2_ → 2Cu^2+^ + 2Fe^3+^ + 2SO_4_^2−^ + 2SO_3_^2−^ + 2CH_3_COO^–^ + 16H_2_O

### 3.5 The effect of leaching time

As shown in Fig. 5e and f, the Fe recovery rate initially declined with leaching time, then increased. In contrast, the Cu recovery rate exhibited a continuously positive correlation with the leaching time, with the rate of change (*k*) increasing from 0.16 h^−1^ between 3 and 4 h to 0.47 h^−1^ between 4 and 5 h. Both phenomena indicate that the concentration of certain reagents was initially depleted and then later restored. Because excess amounts of both CH_3_COOH and H_2_O_2_ were used during leaching, and were found to remain in the solution afterward, their concentrations cannot account for the observed trends. Instead, it is proposed that radicals generated by H_2_O_2_ dissociation significantly contribute to CuFeS_2_ leaching and drive the observed trend. Alone, however, this Taguchi analysis is insufficient to explain such a radical leaching mechanism with respect to time; thus, further validation experiments were performed.

The existence of radicals has been demonstrated in similarly oxidative systems applied to CuFeS_2_, in which Fenton or Fenton-like reactions are the primary source of radicals *via* H_2_O_2_ dissociation.^23,24,44^ Furthermore, recent publications have reported that direct interaction between CuFeS_2_ and H_2_O_2_ can promote radical generation.^45^ Phenol red (a dye commonly used to investigate the action of radical species) is resistant to direct oxidation by H_2_O_2_,^46^ but is rapidly degraded by radical species, such as those generated *via* Fenton (or Fenton-like) reactions. To experimentally validate the role of radical species in chalcopyrite leaching, the degradation of phenol red was monitored using UV-vis spectroscopy. As a commonly used pH indicator, phenol red appears yellow under acidic conditions (pH < 6.8), orange/red under neutral conditions (6.8 < pH < 8.2), and pink/fuchsia under alkaline conditions (pH > 8.2). Once degraded, however, the characteristic absorptions peaks (appearing at 430 or 555 nm, depending upon pH)^46^ disappear, resulting in a colourless solution.

As illustrated in Fig. S8, no significant loss of colouration was observed when phenol red was mixed with either H_2_O_2_ or chalcopyrite leachate individually, though a redshift in the absorption peak was observed during leachate-only treatment, resulting from coordination of the dye to solvated ferric ions.^47,48^ Upon simultaneous addition of H_2_O_2_ and leachate, however, the solution rapidly became colourless. This is further reflected in this sample's featureless UV-vis spectrum (Fig. S9), supporting the conclusion that Fenton (or Fenton-like) reactions drive the increased leaching rate of metals from chalcopyrite during longer leaching times.

Besides aiding in breaking the metal–sulfide bonds in the CuFeS_2_, oxidizing agents can also oxidize sulfide ions in the leaching reaction, thereby preventing them from re-precipitating metal ions. Inspired by this, the leaching process can be divided into three stages. In the first stage, occurring during the first 3 h of leaching, H_2_O_2_ concentration principally controls the dynamics of the leaching reaction. As a result, the metal–sulfide bond cleavage rate (*ν*_bond−cleavage_) is slower than the sulfide-oxidation rate (*ν*_sulfide−oxidation_), thereby guaranteeing that the metal ions remain dissolved. Additionally, H_2_O_2_ also interacted with CuFeS_2_, leached Cu ions, and leached Fe ions to generate radicals. These reactions result in a declining concentration of H_2_O_2_, thereby lowering its activity in the solution. Though the resulting radicals possess a greater intrinsic oxidative activity than H_2_O_2_, their concentration is initially very low, and their influence on the leaching is not yet predominant.

In the second stage (3 to 4 h), the increased concentration of Cu and Fe ions boosts the radical concentration, thus strengthening the influence of radicals on leaching and further weakening the activity of H_2_O_2_. However, the concentration of radicals between 3 and 4 h is limited, being enough to break the metal-sulfide bonds but not to oxidize all the released sulfide ions. This eventually results in *ν*_bond−cleavage_ occurring more rapidly than *ν*_sulfide−oxidation_. As a result, some released sulfide ions remain unoxidized in the solution, causing the re-precipitation of leached Cu and Fe ions. Considering the higher Fe content in the ore, the collision frequency of Fe with sulfide ions is higher than that of Cu with sulfide ions; thus, this negative impact of insufficient oxidation activity is stronger and more obvious for Fe than for Cu.

In the third stage (4 to 5 h), the concentration of Cu ions gradually increases, amplifying the catalytic dissociation of H_2_O_2_ and promoting the generation of radicals. Ultimately, the activity of radicals exceeds the activity of H_2_O_2_ in the leaching reaction, enabling radicals to fulfill the role of primary oxidant in the leaching system. At this point, radicals dominate the leaching process. They ensure the *ν*_bond−cleavage_ is slower than the *ν*_sulfide−oxidation_ while making CuFeS_2_, as well as precipitated Fe and Cu ions, dissolve more readily. Overall, this leads to a significant increase in both Cu and Fe recovery rates between 4 and 5 h.

### 3.6 The effect of milling speed on the ore

**Fig. 7:**
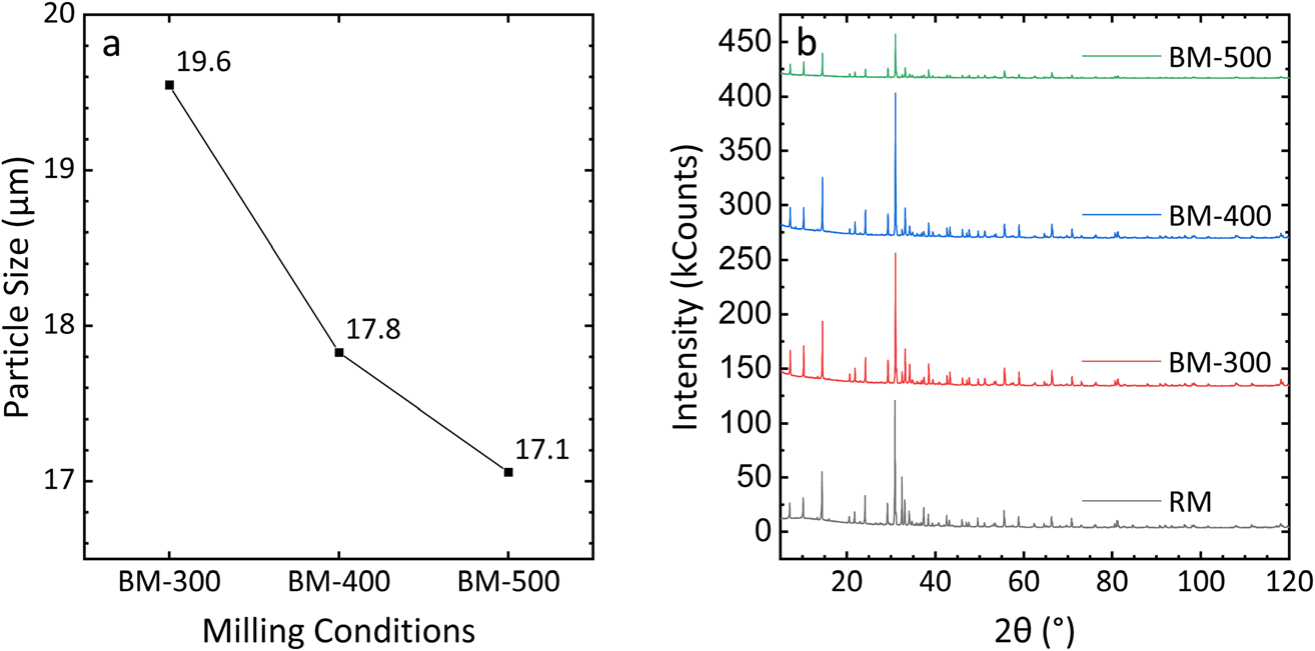
The effect of milling speed on the (a) particle size and (b) the crystal structure of the ore sample.

Before discussing the effect of ball milling on leaching, the impact of milling speed on the ore itself should be examined. The average particle size is shown in Fig. 7a. As the milling speed increases, the average particle size is reduced, with the difference between BM-400 and BM-500 particles being smaller than that between BM-400 and BM-300. As discussed in the introduction, in addition to reducing particle diameter *via* attrition, ball milling can also introduce structural/surface defects to the sample, potentially increasing its susceptibility to leaching. Therefore, it is essential to ascertain whether such effects exist in the present method.

Fig. 7b shows the comparison of the PXRD patterns of raw mineral ore (labeled “RM”) and ball-milled ore. Diffraction patterns from BM-300 and BM-400 particles were similar to those of as-received ores, suggesting the major outcome under these conditions was reduction in particle size. BM-500 particles still maintained sharp characteristic peaks, though the intensity of each peak was lower than that in the as-obtained ores. This emphasizes that BM-500 particles largely retain their crystalline structure, but some defects (*e.g.*, surface defects) do form in their structure, reducing their long-range crystalline ordering.

Rietveld refinement (Fig. S7) further revealed that the proportion of FeS_2_ increased after grinding; however, the proportions of CuFeS_2_ and ZnS did not exhibit a significant change. Additionally, the proportion of muscovite and clinochlore decreased after the milling treatment. The changing of the proportion of those compounds is not significant compared with the inherent systematic error. Therefore, it can be safely concluded that the difference is within acceptable errors.

### 3.7 The effect of milling speed on metal recovery performance

As illustrated in Fig. 5g and h, both Cu and Fe recovery performance demonstrate a negative correlation with the ball milling speed. As discussed in the preceding section, this observation could also be related to the exothermic nature of the leaching reaction and the low thermal stability of H_2_O_2_. As the particle size goes down and defects start forming in the ore structure, the reactivity of the CuFeS_2_ surface increases, triggering a more vigorous reaction. Considering the low stability of H_2_O_2_ at high temperatures, the concentration of H_2_O_2_ would then decrease faster as particle size goes down and/or defects form. Drawing from the preceding section, we know that CuFeS_2_ leaching is less effective under a H_2_O_2_-deficient environment. Therefore, the Cu and Fe recovery performance presents an intuitive inverse correlation with ball-milling speed.

Although the metal recovery rate decreased as the grinding speed increased, pre-processing ores with ball milling was still necessary. Based on the preliminary result obtained by using energy dispersive spectroscopy (EDS), only a 14 ± 2% Cu recovery rate was achieved when using un-milled ores at RT for 4 h, while a 27 ± 2% Cu recovery rate was achieved when using BM-500 particles run under the same reaction conditions. Notably, since the EDS is a surface-sensitive technique, the recovery rate obtained from EDS will be less accurate than the result we reported in ICP-OES. However, this 93% increase in Cu recovery performance can effectively highlight the importance of ball milling treatment in efficient CuFeS_2_ leaching.

### 3.8 Extending the CH_3_COOH/H_2_O_2_ system to other minerals

It is also useful to consider the feasibility of the CH_3_COOH/H_2_O_2_ combination in leaching other natural minerals. Taking advantage of the presence of ZnS in the ore, the performance of Zn leaching was also assessed. Based on the result (shown in Fig. S10 and Table S1), the highest Zn recovery rate is 96 ± 1% when using BM-300 particles and 10 mL of 17 M CH_3_COOH at 40 °C for 5 h. It was also found that the response of Zn to each influence factor was similar to that of Fe, as shown in Fig. S11. As a result, it is very likely that this CH_3_COOH/H_2_O_2_ leaching system will be useful in extracting metals from a diverse range of minerals.

## 4. Discussion

Experimental observations revealed that each component of the CH_3_COOH/H_2_O_2_ leaching system played multiple roles. While the broader role of CH_3_COOH in hydrometallurgy remains a subject of some debate, experimental results presented in this work reveal some critical information regarding its function in the CH_3_COOH/H_2_O_2_ system. First, it plays an important role as a proton donor, as shown by the preliminary leaching results presented in Fig. 1. In this role, it facilitates protonation of sulfide groups at the ore surface, aiding in cleavage the metal-sulfide bond and oxidation of sulfur; thus, it ensures a continuous supply of exposed metal ion sites for further leaching. Second, the acidity of CH_3_COOH also helps ensure consistently acidic conditions (pH ≈ 1) to stabilize Fe^2+^/Fe^3+^ ions in solution, as a pH greater than 3 induces precipitation of FeOOH, which could deposit onto the ore surface and inhibit further leaching. Third, and equally critical is the role of acetate in complex formation with Fe ions. This interaction was revealed by the characteristic red-brown colour of the leaching solution (Fig. S12), reflecting the formation of Fe^3+^–acetate complexes (*e.g.*, [Fe_3_O(CH_3_COO)_6_(H_2_O)_3_]^+^). This complexation lowers the concentration (and, thus, chemical activity) of free ions such as Fe^3+^ and Cu^2+^ in the system, thereby shifting the reaction toward the CuFeS_2_ dissolution in accordance with Le Chatelier's principle.

Apart from its initial role as an oxidizing agent, H_2_O_2_ contributes additional functionality by catalytically dissociating to form radicals that promote the more rapid leaching of CuFeS_2_. These complex interactions, including radical generation and varying rates of reaction, cannot be fully explained by the results of Taguchi analysis alone; however, the key role of radical species is clearly supported by phenol red dye degradation experiments, highlighting the importance of Fenton and Fenton-like reactions in driving high metal recovery yields.

To provide a clearer demonstration of the method's superior performance, Table 5 provides a detailed comparison against several recent reported approaches. Given that Cu in the as-obtained ore is present only in CuFeS_2_, the Cu recovery rate was used to make this comparison. The proposed CH_3_COOH/H_2_O_2_ system outperforms other recently developed methods in both leaching efficiency and environmental friendliness, demonstrating its potential as a more sustainable alternative for practical applications. In addition, the CH_3_COOH/H_2_O_2_ leaching system can achieve full Cu recovery under ambient conditions, compared with other studies. This property could significantly lower the carbon footprint associated with heat treatment and eliminate the thermal risk of the instrument and chemicals, fulfilling the current expectations of building a sustainable society. Moreover, the leaching duration is only 5 h, which is a substantial improvement over previous processes that typically required several tens of hours.

**Table 5:** Comparative analysis of study findings with existing literature

Author	Year	Reagent	Leaching temperature (°C)	Leaching duration (h)	Cu recovery rate (%)
**This study**	**2025**	**CH**_**3**_ **COOH**	**RT (∼20)**	**5**	**100**
		**H**_**2**_ **O**_**2**_			
Dakkoune *et al.*^7^	2023	H_2_O_2_	42	216	85
		H_2_SO_4_			
		Glass beads			
Liu *et al.*^51^	2023	HCl	85	15	95
		FeCl_3_			
		Tartrate			
Li *et al.*^52^	2019	FeCl_3_ (CH_2_OH)_2_	90	24	100
Zhong & Li^53^	2019	H_2_SO_4_	75	150	100
		NaCl			
Solís-Marcial & Lapidus^29^	2014	H_2_SO_4_	40	5	65
		CH_3_COOH			
		O_3_			
		CuSO_4_			

The CH_3_COOH/H_2_O_2_ leaching system reduces hazardous emissions relative to traditional H_2_SO_4_ methods, as it produces much lower concentrations for sulfates in the resulting aqueous waste (due to the absence of H_2_SO_4_ as a reagent); by comparison, acetic acid is both biodegradable and a significantly weaker acid (p*K*_a_ = 4.75) than H_2_SO_4_ (p*K*_a_ < 0), resulting in less-acidic tailings material that can be more easily remediated. While concentrated CH_3_COOH still poses chemical hazards, safe handling procedures are already available, due to its widespread use in industrial processes ranging from plastic manufacturing to food preservation.^49,50^ (For example, vinyl acetate monomer, a common polymer synthesis material, is synthesized exclusively using concentrated CH_3_COOH rather than its dilute form.^50^)

However, while the toxicity of acetic acid is lower than that of strong acids (*e.g.*, H_2_SO_4_), its use at high (17 M) concentrations warrants certain precautions. This is especially true if this reaction is to be performed on significantly larger scales. As indicated by its Safety Data Sheet, acetic acid is a severe corrosive (Category 1A), posing a risk of damage to body tissues, mucous membranes, and vulnerable materials (metals, plastics, *etc.*). As such, small quantities should be handled while wearing protective gloves (nitrile, neoprene, or butyl rubber, with verified break-through times), proper eye protection (*e.g.*, laboratory goggles), and protective clothing (*e.g.*, flame-resistant lab coat), and kept away from acid-sensitive materials. Handling large volumes (tens to hundreds of litres) requires the use of additional respiratory protections, such as an acid gas respirator and goggles, to prevent acute eye damage and/or respiratory distress. As it is combustible (Category 3c, flash point: 40 °C), acetic acid should be maintained in a well-ventilated environment away from sources of heat and ignition.

While the mechanical ball milling process employed in this study plays an important role in activating the ore material and reducing solvent waste, it does represent a significant input of energy. At the laboratory scale, a very rough estimate of the specific energy consumption associated with planetary ball milling results in an astronomical estimate of *ca.* 390 000 kWh t^−1^, economy of scale means that energy consumption during industrial-scale ball milling of such materials is closer to 15–150 kWh ton^−1^.^54^ According to a systematic analysis by Celis *et al.*, transitioning to an industrial-grade setup reduces energy requirements to 150 kWh t^−1^ (batch ball milling test) or 18 kWh ton^−1^ (continuous ball milling test) while obtaining a similar chalcopyrite particle size, thereby consistent with the commercial feasibility standards.^55^ Even at these more realistic levels of energy consumption, it would be necessary to weight the advantages off ball milling (*i.e.*, increased metal recovery) against the additional required energy consumption.

In terms of material cost, the substitution of CH_3_COOH for H_2_SO_4_ is unlikely to offer an immediate cost advantage. At the 200 L scale, CH_3_COOH is approximately twice the cost of H_2_SO_4_ (39 CAD L^−1^*versus* 17 CAD L^−1^) at similar level of purity (ACS Reagent Grade); however, at industrial scales, the increasingly lower cost of sulfuric acid leads to a 4- to 5-times greater cost for acetic acid (638 USD t^−1^*versus* 137 USD t^−1^). Furthermore, hydrogen peroxide costs come in even higher at 921 USD t^−1^, resulting in considerably higher consumable costs relative to simple sulfuric acid. At best, an argument might be made, that this material cost could be at least partially offset by the less-toxic nature of CH_3_COOH and H_2_O_2_, thereby reducing the need for such heightened safety equipment and measures as necessitated by H_2_SO_4_, though a full technoeconomic assessment would be required to accurately quantify such factors. Combined with reduced risk to infrastructure and facility workers handling such materials, it is plausible that the CH_3_COOH/H_2_O_2_ system could be an appealing option in a sufficiently safety/environment-conscious setting but comes at a higher financial cost.

Unfortunately, greener reagents do not necessarily translate into a green leaching process. It is necessary to consider downstream purification and waste treatment should be considered. Increasing the pH of the waste stream is a common method or preventing acidification of the local environment and should also help to precipitate metal ions, thereby decreasing their concentration (and toxicity). Highly soluble metal ions could be further removed *via* chelation/adsorption and physical separation from solution. Fortunately, CH_3_COOH is biodegradable; therefore, neutralization and bioremediation of waste leachate could be considered. Additionally, while sulfates and sulfites are likely to be present, the absence of H_2_SO_4_ as a leaching agent means that they would be present in significantly lower concentrations, thereby reducing their environmental impact.

Collectively, the combination of low-toxicity compounds, minimized waste, and energy-saving operation underscores that the presented method firmly aligns with the 12 Principles of Green Chemistry, particularly emphasizing waste prevention, energy efficiency, less hazard, and safer solvents. The proposed strategic modification enhances the environmental compatibility of the hydrometallurgical process while maintaining a high metal recovery rate and efficiency.

## 5. Conclusion

In conclusion, the applicability of a CH_3_COOH/H_2_O_2_ leaching system has been corroborated in this study by characterizing both ore residues and leachate under a wide range of conditions and using orthogonal (Taguchi) experimental design. This method could offer a safer alternative to the use of strong oxidizing acids, such as sulfuric acid, in hydrometallurgy. Additionally, neither the slowing down of the leaching reaction nor the formation of passivation layers on the ore surface were observed using these reagents. Optimal leaching conditions were found for the leaching temperature, leaching time, ball milling speed, and volume of CH_3_COOH. Mixing 1.5 g BM-300 particles with 15 mL of 17 M CH_3_COOH and 15 mL of 30 wt% H_2_O_2_ for 5 h at RT resulted in a nearly 100% Cu recovery rate while mixing 1.5 g BM-300 particles with 10 mL of 17 M CH_3_COOH, 5 mL of distilled water, and 15 mL of 30 wt% of H_2_O_2_ for 5 h at 40 °C could realize a Fe recovery rate of *ca.* 60%.

Furthermore, the leaching temperature was found to alter the leaching reaction by influencing the behavior of FeS_2_ and H_2_O_2_. The leaching time effect highlighted that radicals, generated by H_2_O_2_, joined the leaching reaction and played a significant role. Moreover, the CH_3_COOH volume effect could be explained by Le Chatelier's Principle. Finally, while some ball milling treatment was beneficial (affording a 93% increase in Cu recovery rate), higher ball milling speeds reduced the particle size and introduced defects to the original crystal structure, to the point that they negatively impacted Cu and Fe recovery rates.

Future work will focus on fully identifying a more comprehensive mechanism and testing the feasibility of the CH_3_COOH/H_2_O_2_ leaching system on other minerals. Ultimately, this work contributes to a deeper understanding of hydrometallurgical leaching, particularly in CuFeS_2_, and offers valuable insights into means of advancing green chemistry without sacrificing chemical efficacy.

## Author contributions

Z. F. performed all experiments, analysed the resulting data, and drafted this manuscript. Z. F. and P. N. D. conceptualized the study, contributed to document editing and revision, and approved the final version for publication. P. N. D. supervised Z. F. and provided laboratory resources and funding.

## Conflicts of interest

The authors declare no competing financial interest.

## Supplementary Material

RA-016-D6RA00565A-s001

## Data Availability

Data presented used in this work is available on ChemRXIV. Supplementary information (SI): additional X-ray diffraction patterns, supporting spectra, and illustrative images. See DOI: https://doi.org/10.1039/d6ra00565a.

## References

[cit1] Yang H., Zhao S., Wang G., Zhang Q., Jin Z., Tong L., Chen G., Qiu X. (2022). Mechanical Activation Modes of Chalcopyrite Concentrate and Relationship between Microstructure and Leaching Efficiency. Hydrometallurgy.

[cit2] Ren Z., Chao C., Krishnamoorthy P., Asselin E., Dixon D. G., Mora N. (2022). The Overlooked Mechanism of Chalcopyrite Passivation. Acta Mater..

[cit3] Eksteen J. J., Oraby E. A., Tanda B. C. (2017). A Conceptual Process for Copper Extraction from Chalcopyrite in Alkaline Glycinate Solutions. Miner. Eng..

[cit4] Barton I. F., Hiskey J. B. (2022). Chalcopyrite Leaching in Novel Lixiviants. Hydrometallurgy.

[cit5] Tian Z., Li H., Wei Q., Qin W., Yang C. (2021). Effects of Redox Potential on Chalcopyrite Leaching: An Overview. Miner. Eng..

[cit6] Nabizadeh A., Aghazadeh V. (2015). Dissolution Study of Chalcopyrite Concentrate in Oxidative Ammonia/Ammonium Carbonate Solutions at Moderate Temperature and Ambient Pressure. Hydrometallurgy.

[cit7] Dakkoune A., Bourgeois F., Po A., Joulian C., Hubau A., Touzé S., Julcour C., Guezennec A.-G., Cassayre L. (2023). Hydrometallurgical Processing of Chalcopyrite by Attrition-Aided Leaching. ACS Eng. Au.

[cit8] Li Y., Kawashima N., Li J., Chandra A. P., Gerson A. R. (2013). A Review of the Structure, and Fundamental Mechanisms and Kinetics of the Leaching of Chalcopyrite. Adv. Colloid Interface Sci..

[cit9] Nesse W. D. , Introduction to Optical Mineralogy, 9. printing, Oxford University Press, Oxford, 2nd edn, 1991

[cit10] Mu Y., Peng Y., Lauten R. A. (2018). The Galvanic Interaction between Chalcopyrite and Pyrite in the Presence of Lignosulfonate-Based Biopolymers and Its Effects on Flotation Performance. Miner. Eng..

[cit11] Moskalyk R. R., Alfantazi A. M. (2003). Review of Copper Pyrometallurgical Practice: Today and Tomorrow. Miner. Eng..

[cit12] Niu J., Liu H., Jin Y., Fan B., Qi W., Ran J. (2022). Comprehensive Review of Cu-Based CO2 Hydrogenation to CH3OH: Insights from Experimental Work and Theoretical Analysis. Int. J. Hydrogen Energy.

[cit13] Zhao H., Yu R., Ma S., Xu K., Chen Y., Jiang K., Fang Y., Zhu C., Liu X., Tang Y., Wu L., Wu Y., Jiang Q., He P., Liu Z., Tan L. (2022). The Role of Cu1–O3 Species in Single-Atom Cu/ZrO2 Catalyst for CO2 Hydrogenation. Nat. Catal..

[cit14] Crundwell F. K. (1988). The Influence of the Electronic Structure of Solids on the Anodic Dissolution and Leaching of Semiconducting Sulphide Minerals. Hydrometallurgy.

[cit15] Medina Ferrer F., Dold B., Jerez O. (2021). Dissolution Kinetics and Solubilities of Copper Sulfides in Cyanide and Hydrogen Peroxide Leaching: Applications to Increase Selective Extractions. J. Geochem. Explor..

[cit16] Spooren J., Binnemans K., Björkmalm J., Breemersch K., Dams Y., Folens K., González-Moya M., Horckmans L., Komnitsas K., Kurylak W., Lopez M., Mäkinen J., Onisei S., Oorts K., Peys A., Pietek G., Pontikes Y., Snellings R., Tripiana M., Varia J., Willquist K., Yurramendi L., Kinnunen P. (2020). Near-Zero-Waste Processing of Low-Grade, Complex Primary Ores and Secondary Raw Materials in Europe: Technology Development Trends. Resour. Conserv. Recycl..

[cit17] Romero R., Mazuelos A., Palencia I., Carranza F. (2003). Copper Recovery from Chalcopyrite Concentrates by the BRISA Process. Hydrometallurgy.

[cit18] Yang D., Kirke M., Fan R., Priest C. (2019). Investigation of Chalcopyrite Leaching Using an Ore-on-a-Chip. Anal. Chem..

[cit19] Baba A. A., Ghosh M. K., Pradhan S. R., Rao D. S., Baral A., Adekola F. A. (2014). Characterization and Kinetic Study on Ammonia Leaching of Complex Copper Ore. Trans. Nonferrous Met. Soc. China.

[cit20] Rasouli A. , Copper Extraction from Chalcopyrite through a Two-step Non-Oxidative/Oxidative Leaching Process, PhD thesis, Queen's University, 2023

[cit21] Dreisinger D., Abed N. (2002). A Fundamental Study of the Reductive Leaching of Chalcopyrite Using Metallic Iron Part I: Kinetic Analysis. Hydrometallurgy.

[cit22] Mahajan V., Misra M., Zhong K., Fuerstenau M. C. (2007). Enhanced Leaching of Copper from Chalcopyrite in Hydrogen Peroxide–Glycol System. Miner. Eng..

[cit23] Ruiz-Sánchez A., Lapidus G. T. (2022). A Study to Understand the Role of Ethylene Glycol in the Oxidative Acid Dissolution of Chalcopyrite. Miner. Eng..

[cit24] De Laat J., Gallard H. (1999). Catalytic Decomposition of Hydrogen Peroxide by Fe(III) in Homogeneous Aqueous Solution: Mechanism and Kinetic Modeling. Environ. Sci. Technol..

[cit25] Wang X., Wu W., Zhang L., Fu L., Li X. (2022). Preparation of One-Part Alkali-Activated Nickel Slag Binder Using an Optimal Ball Milling Process. Constr. Build. Mater..

[cit26] Blanc N., Mayer-Laigle C., Frank X., Radjai F., Delenne J.-Y. (2020). Evolution of Grinding Energy and Particle Size during Dry Ball-Milling of Silica Sand. Powder Technol..

[cit27] Priestley I., Battilocchio C., Iosub A. V., Barreteau F., Bluck G. W., Ling K. B., Ingram K., Ciaccia M., Leitch J. A., Browne D. L. (2023). Safety Considerations and Proposed Workflow for Laboratory-Scale Chemical Synthesis by Ball Milling. Org. Process Res. Dev..

[cit28] Nikkhou F., Xia F., Knorsch M., Deditius A. P. (2020). Mechanisms of Surface Passivation during Galena Leaching by Hydrogen Peroxide in Acetate and Citrate Solutions at 25–50 °C. ACS Sustain. Chem. Eng..

[cit29] Solís-Marcial O. J., Lapidus G. T. (2014). Study of the Dissolution of Chalcopyrite in Sulfuric Acid Solutions Containing Alcohols and Organic Acids. Electrochim. Acta.

[cit30] Chen H., He J., Zhu L., Liu B., Zhou K., Xu J., Guo C. (2022). Eco-Friendly Oxidation Leaching from Chalcopyrite Powder and Kinetics Assisted by Sodium Chloride in Organic Acid Media. Adv. Powder Technol..

[cit31] Radhakrishnan J., Sridhar S., Zuber M., Ng E. Y. K., B S. S. (2023). Design Optimization of a Contra-Rotating VAWT: A Comprehensive Study Using Taguchi Method and CFD. Energy Convers. Manag..

[cit32] Shojaei S., Shojaei S., Band S. S., Farizhandi A. A. K., Ghoroqi M., Mosavi A. (2021). Application of Taguchi Method and Response Surface Methodology into the Removal of Malachite Green and Auramine-O by NaX Nanozeolites. Sci. Rep..

[cit33] Zhang J. Z., Chen J. C., Kirby E. D. (2007). Surface Roughness Optimization in an End-Milling Operation Using the Taguchi Design Method. J. Mater. Process. Technol..

[cit34] Degen T., Sadki M., Bron E., König U., Nénert G. (2014). The HighScore Suite. Powder Diffr..

[cit35] Kabekkodu S. N., Dosen A., Blanton T. N. (2024). PDF-5+: A Comprehensive Powder Diffraction File™ for Materials Characterization. Powder Diffr..

[cit36] Fairley N., Fernandez V., Richard-Plouet M., Guillot-Deudon C., Walton J., Smith E., Flahaut D., Greiner M., Biesinger M., Tougaard S., Morgan D., Baltrusaitis J. (2021). Systematic and Collaborative Approach to Problem Solving Using X-Ray Photoelectron Spectroscopy. Appl. Surf. Sci. Adv..

[cit37] Ghahremaninezhad A., Dixon D. G., Asselin E. (2013). Electrochemical and XPS Analysis of Chalcopyrite (CuFeS2) Dissolution in Sulfuric Acid Solution. Electrochim. Acta.

[cit38] Xu S., Zanin M., Skinner W., Brito E Abreu S. (2021). Surface Chemistry of Oxidised Pyrite during Grinding: ToF-SIMS and XPS Surface Analysis. Miner. Eng..

[cit39] Fantauzzi M., Elsener B., Atzei D., Rigoldi A., Rossi A. (2015). Exploiting XPS for the Identification of Sulfides and Polysulfides. RSC Adv..

[cit40] Crundwell F. K., Bryson L. J., Van Aswegen A., Knights B. D. H. (2021). Effect of Chopped Light on the Dissolution and Leaching of Chalcopyrite. Miner. Eng..

[cit41] Antonijević M. M., Dimitrijević M., Janković Z. (1997). Leaching of Pyrite with Hydrogen Peroxide in Sulphuric Acid. Hydrometallurgy.

[cit42] Antonijević M. M., Janković Z. D., Dimitrijević M. D. (2004). Kinetics of Chalcopyrite Dissolution by Hydrogen Peroxide in Sulphuric Acid. Hydrometallurgy.

[cit43] Lu Z. Y., Jeffrey M. I., Lawson F. (2000). The Effect of Chloride Ions on the Dissolution of Chalcopyrite in Acidic Solutions. Hydrometallurgy.

[cit44] Barb W. G., Baxendale J. H., George P., Hargrave K. R. (1949). Reactions of Ferrous and Ferric Ions with Hydrogen Peroxide. Nature.

[cit45] Yang K., Zhai Z., Liu H., Zhao T., Yuan D., Jiao T., Zhang Q., Tang S. (2023). Peracetic Acid Activation by Natural Chalcopyrite for Metronidazole Degradation: Unveiling the Effects of Cu-Fe Bimetallic Sites and Sulfur Species. Sep. Purif. Technol..

[cit46] Wang M., Zhang S., Zhao Z., Li Z., Nai J., Liu X., Zhang K., Zhong J., Li Y., Jiang L. (2023). Phenol Red Hydrogel as pH Indicator with Protection against Nanoceria Degradation. J. Sci. Adv. Mater. Devices.

[cit47] Wesp E. F., Brode W. R. (1934). The Absorption Spectra of Ferric Compounds. I. The Ferric Chloride—Phenol Reaction. J. Am. Chem. Soc..

[cit48] Banerjee S., Haldar B. C. (1950). Constitution of Ferri-Phenol Complex in Solution. Nature.

[cit49] Jacobs H. P., Elias W. C., Heck K. N., Jiang S., Dodson J. J., Sandoval-Pauker C., Foucher A. C., Cha B. J., Arredondo J. H., Chen L., Mueller S. G., Alexander S. R., Senftle T. P., Miller J. T., Wong M. S. (2025). Dynamic Behavior of Molecular Pd-Acetate Trimers and Dimers in Heterogeneous Vinyl Acetate Synthesis. Nat. Commun..

[cit50] Zha Z., Deshlahra P. (2021). Mechanistic Framework and Effects of High Coverage in Vinyl Acetate Synthesis. ACS Catal..

[cit51] Liu X.-J., Liao Y., Liu Q., Wu M. (2023). The Effect of Tartrate on the Mild Leaching of Low-Grade Polymetallic Complex Chalcopyrite Ore in Acidic Ferric Chloride Solution. New J. Chem..

[cit52] Li X., Monnens W., Li Z., Fransaer J., Binnemans K. (2020). Solvometallurgical Process for Extraction of Copper from Chalcopyrite and Other Sulfidic Ore Minerals. Green Chem..

[cit53] Zhong S., Li Y. (2019). An Improved Understanding of Chalcopyrite Leaching Kinetics and Mechanisms in the Presence of NaCl. J. Mater. Res. Technol..

[cit54] Chehreh Chelgani S., Fatahi R., Pournazari A., Nasiri H. (2025). Modeling Energy Consumption Indexes of an Industrial Cement Ball Mill for Sustainable Production. Sci. Rep..

[cit55] Celis C., Antoniou A., Cuisano J., Pillihuaman A., Maza D. (2021). Experimental Characterization of Chalcopyrite Ball Mill Grinding Processes in Batch and Continuous Flow Processing Modes to Reduce Energy Consumption. J. Mater. Res. Technol..

