# Supplementary material for: A mild and highly effective method of leaching metal from chalcopyrite using household chemicals
Source: RSC Adv. 2026 Mar 25;16(18):16672–86. doi: 10.1039/d6ra00565a (PMC13014650; doi:10.1039/d6ra00565a)
Supplement: RA-016-D6RA00565A-s001 [file RA-016-D6RA00565A-s001.pdf]

This journal is © The Royal Society of Chemistry 2026

Supplementary

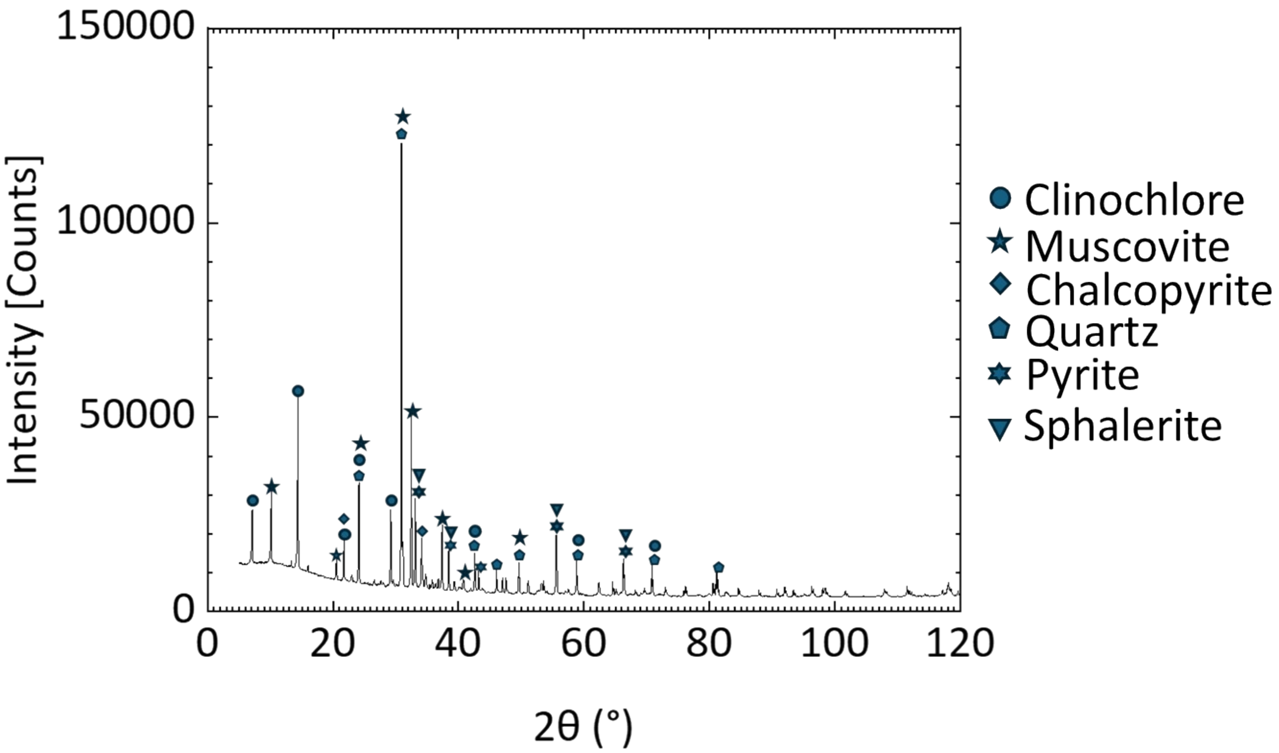

Figure S1. The PXRD pattern of raw Kidd Creek ore. Significant peaks from known components are highlighted.

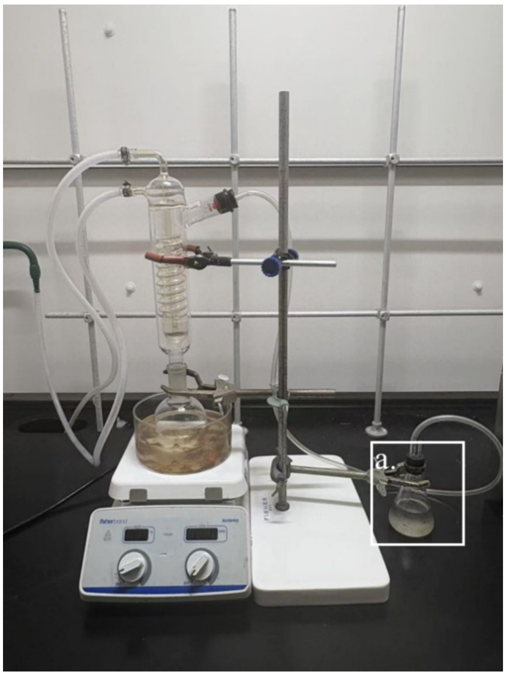

Figure S2. The reactor for $CuFeS_2$ leaching includes a condenser, a 100 mL round-bottom flask (containing H_2O_2, CH_3COOH, distilled water, and ore), and a NaOH safety trap (highlighted in a white frame)

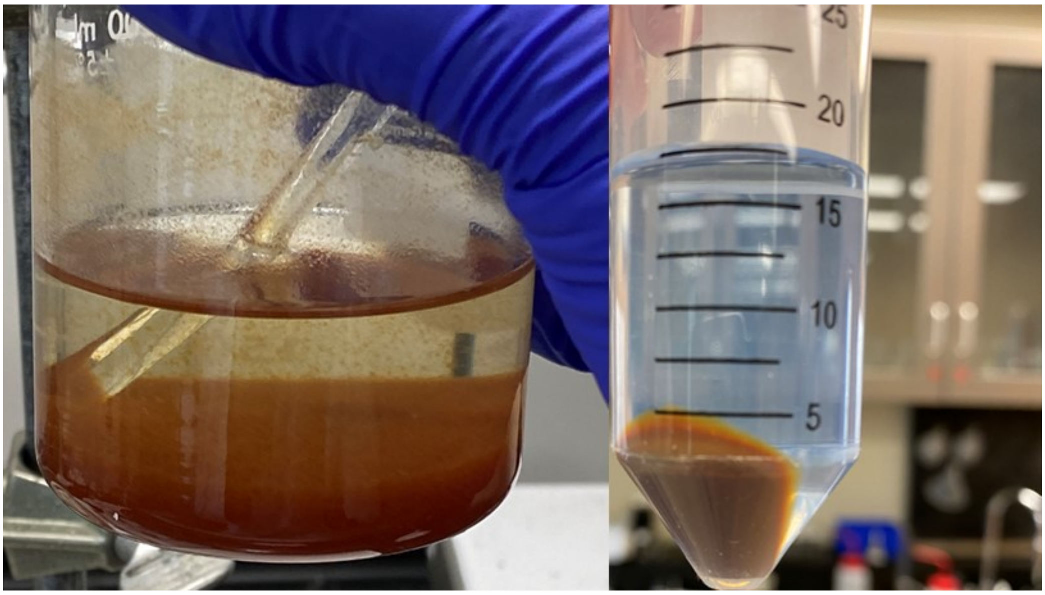

Figure S3. Qualitative clues for successful $CuFeS_2$ leaching

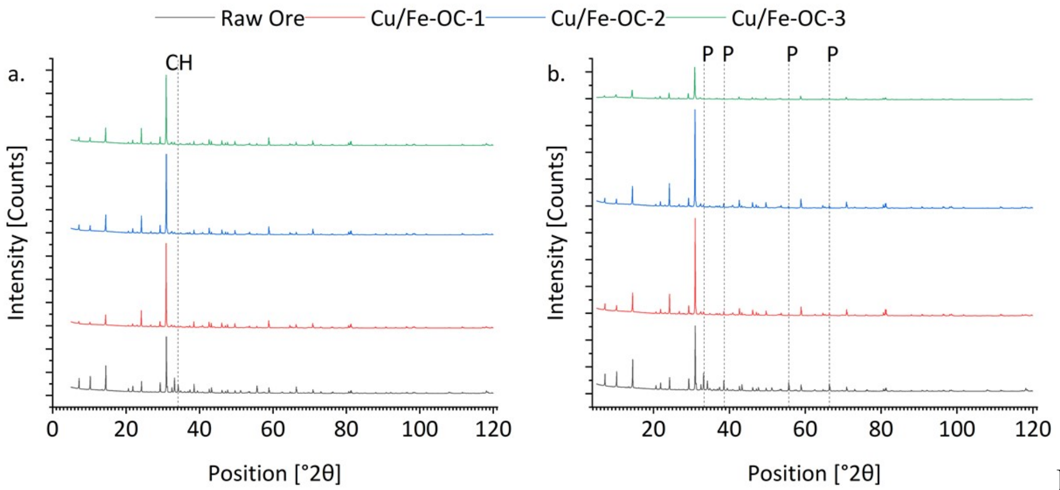

Figure S4. The full-scan PXRD pattern of the ore residue under the optimum leaching conditions of (a) Cu and (b) Fe.

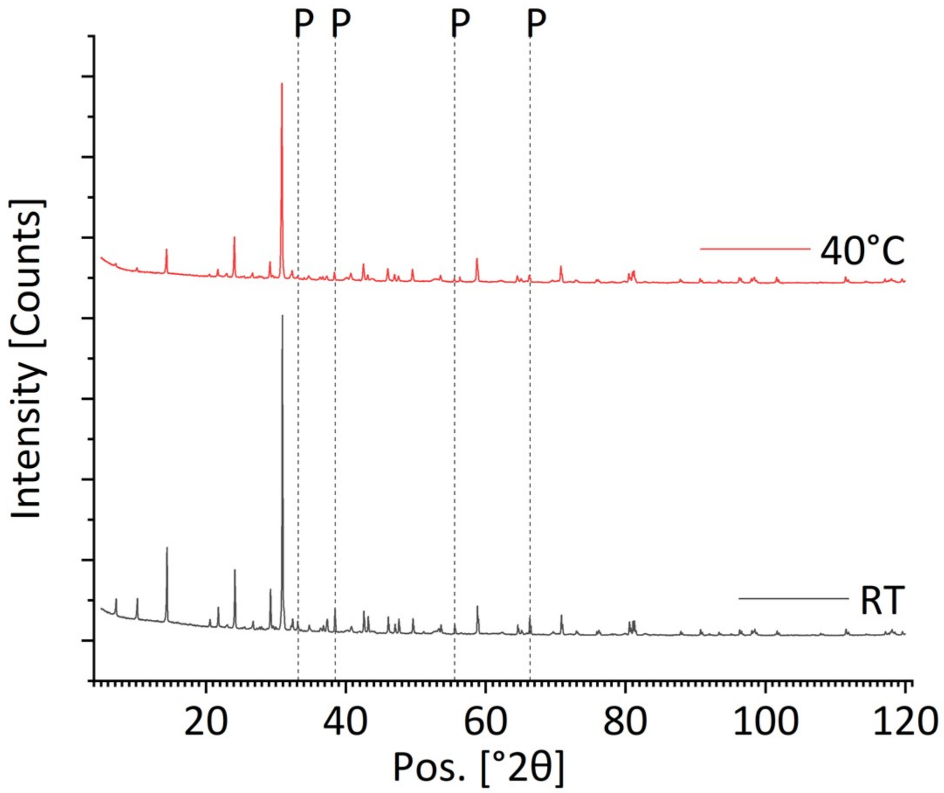

Figure S5. The full-scan PXRD pattern of ore residue at RT and 40 °C

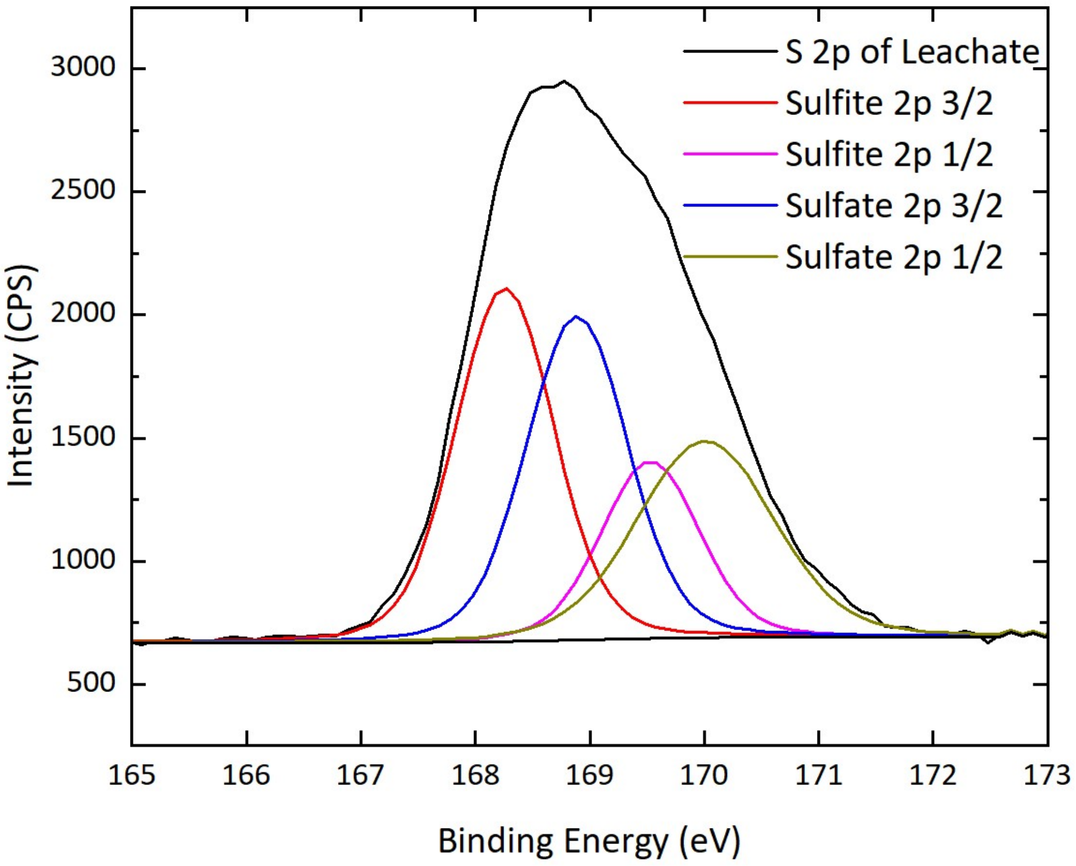

Figure S6. S 2p XPS fitted with each component for leachate.

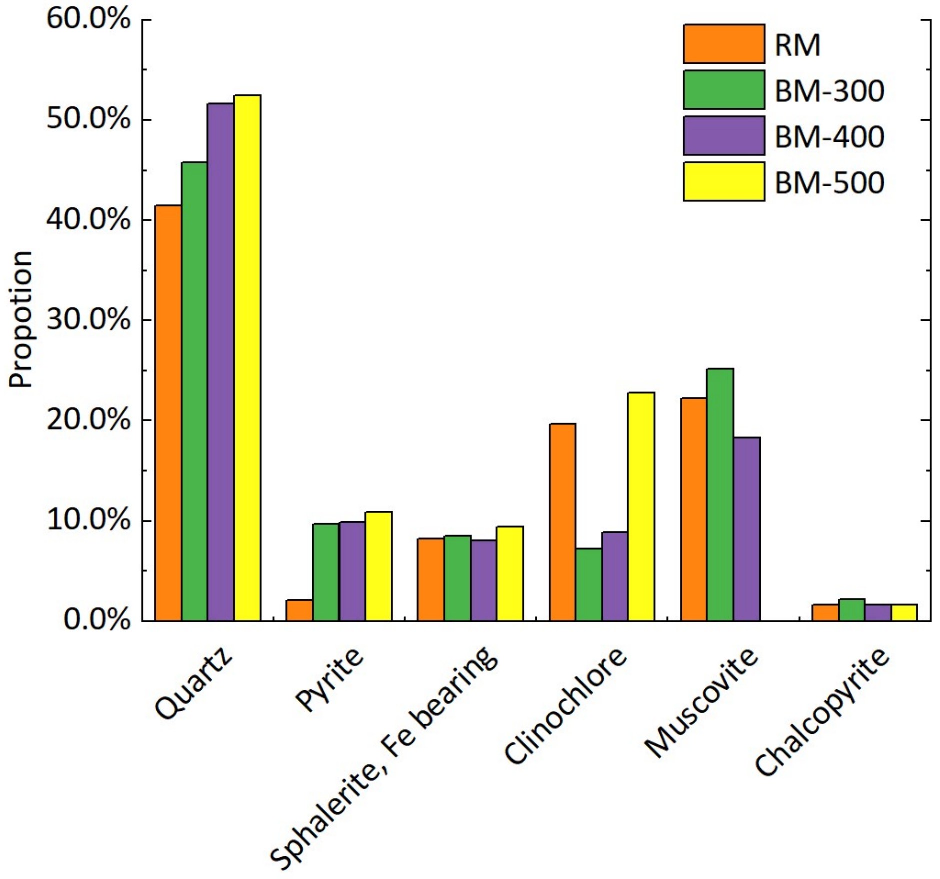

Figure S7. The proportion of each mineral in natural ore and ball-milled ore

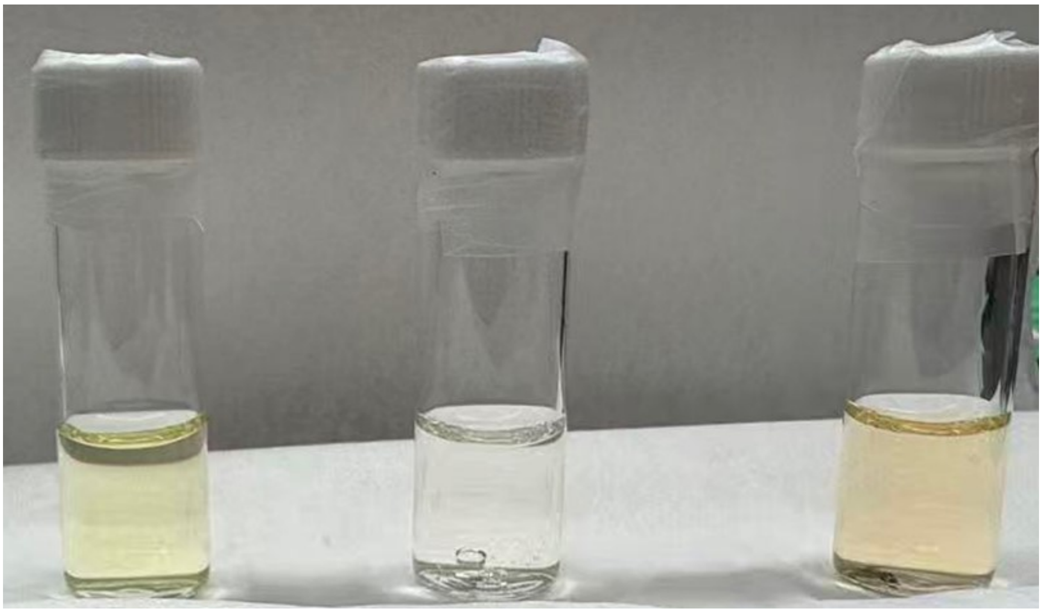

Figure S8. Visual colour changes of Phenol Red dye after introducing chemical agents involved in the generation of oxidative radical species chemicals. From left to right: H_2O_2 only, H_2O_2 + Leachate, and Leachate only.

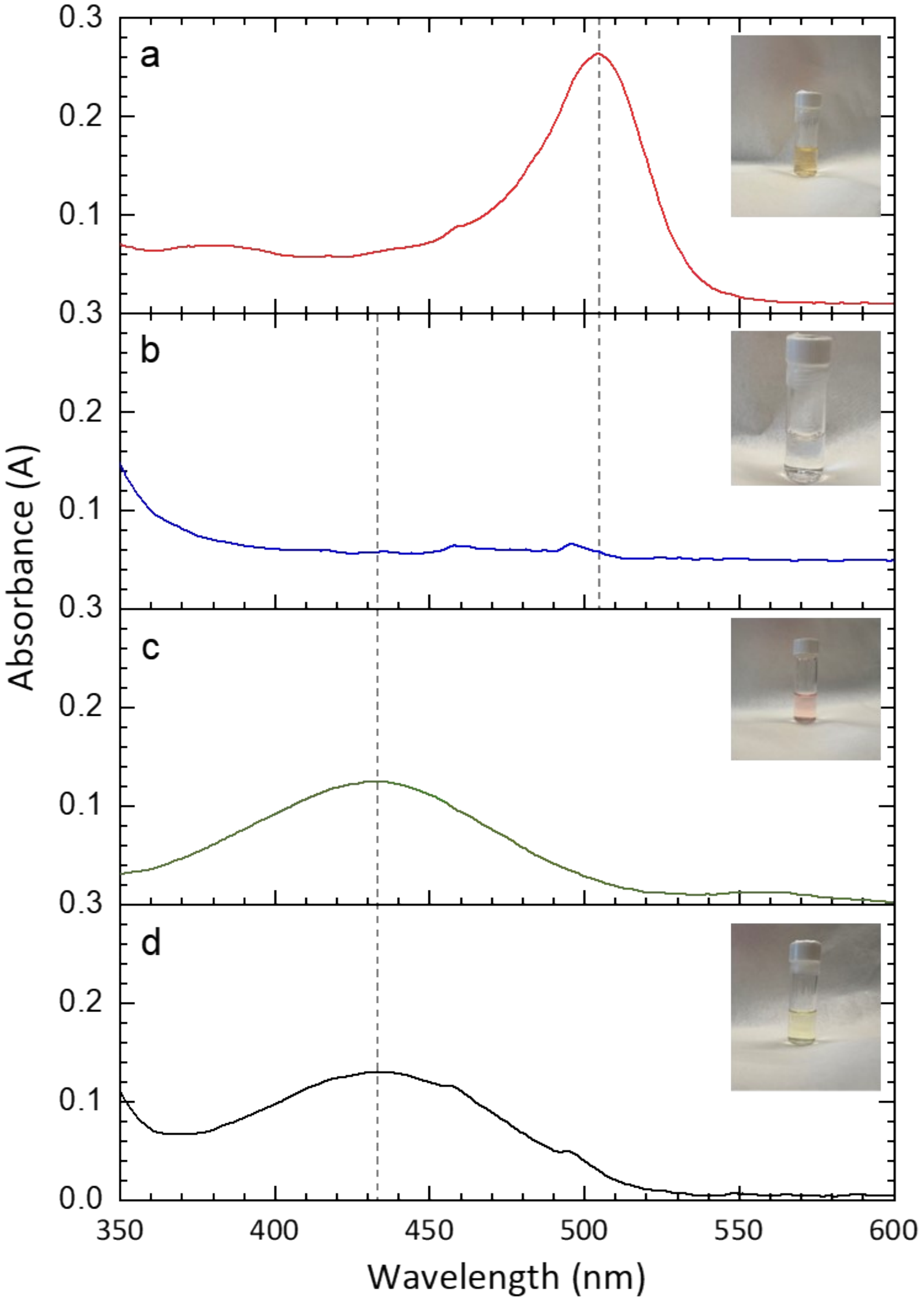

Figure S9. UV-vis absorbance spectra of Phenol Red dye acquired 12 min after treatment with (a) only leachate, (b) leachate and H_2O_2, (c) de-ionized water, or (d) only H_2O_2.

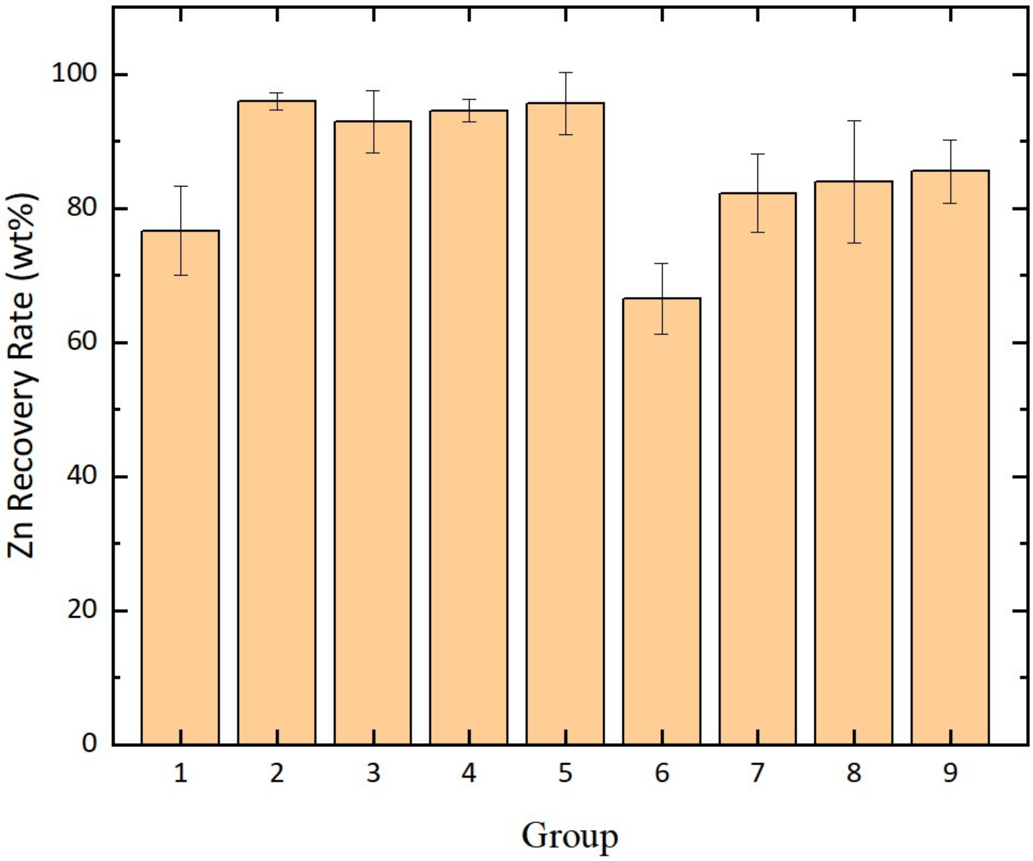

Figure S10. Average Zn recovery rates for each group used in the Taguchi analysis

Level	Milling speed (rpm)	Leaching Temperature (°C)	Volume of Acetic Acid (mL)	Leaching Time (h)
1	38.87	38.47	37.47	38.61
2	38.50	39.21	39.26	38.10
3	38.42	38.12	39.06	39.08
Maximum Value	38.87	39.21	39.26	39.08
Minimum Value	38.42	38.12	37.47	38.10
Range	0.45	1.09	1.78	0.98
Rank	4	2	1	3
Best Condition	BM-300	40	10	5

Table S1. Deep analysis of orthogonal experiments for Zn

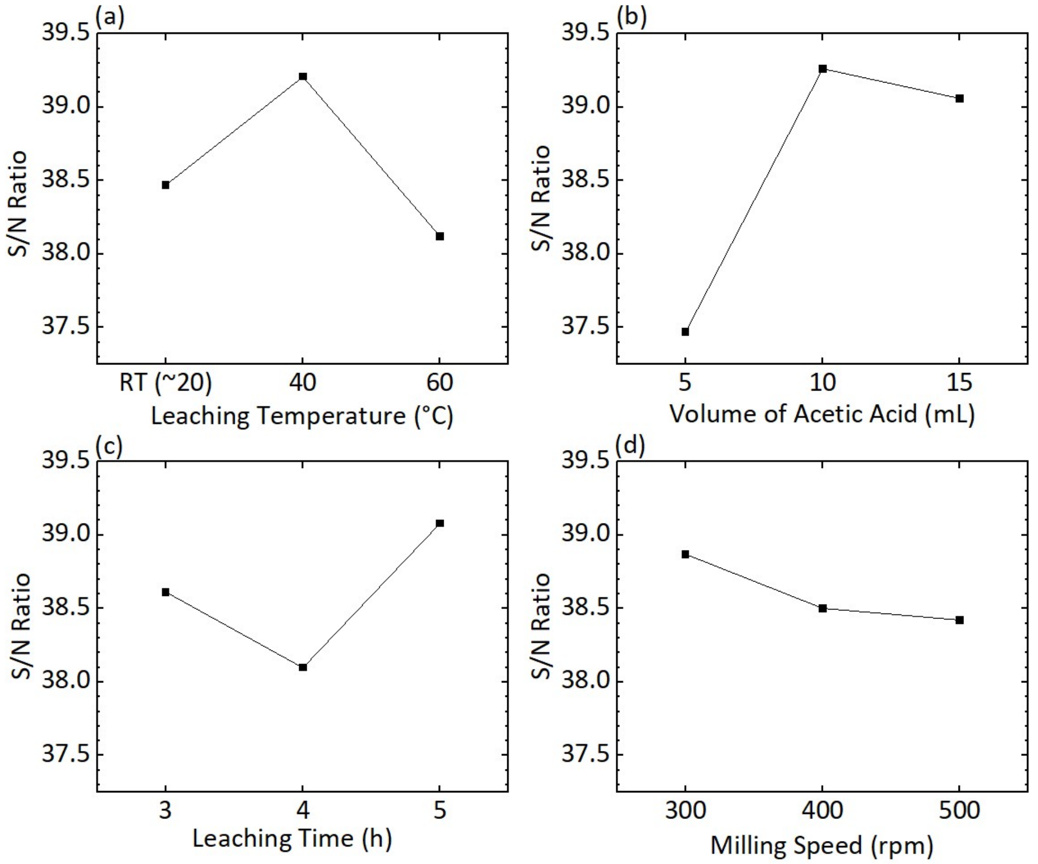

Figure S11. The effect of the leaching temperature (a), CH_3COOH volume (b), the leaching time (c), and the milling speed (d) on Zn

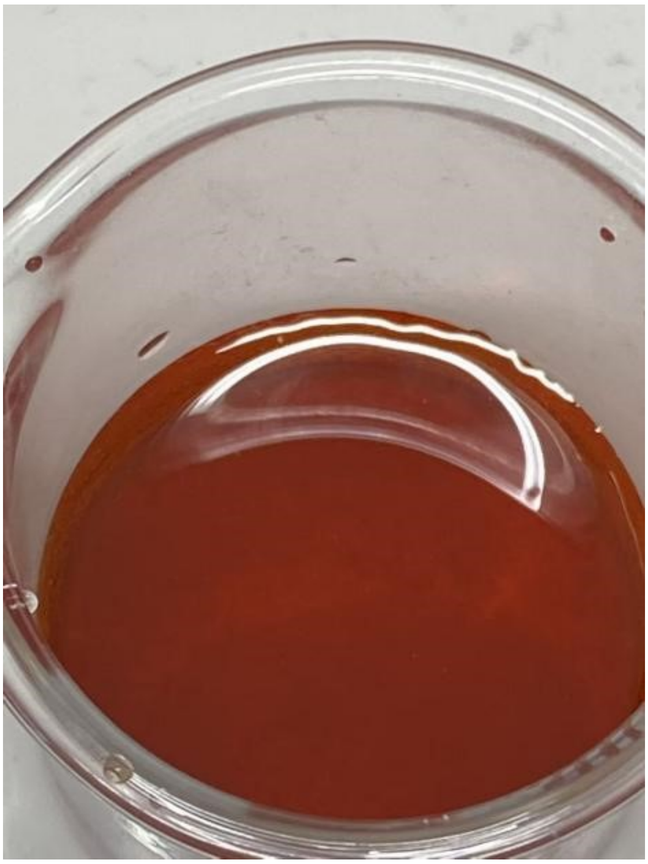

Figure S12. Illustration of the red-orange colour of the leachate solution, resulting from iron acetate formation.
